# Nanoscopic analysis of tight junction organization in in vitro blood-brain barrier models

**DOI:** 10.1186/s12987-026-00799-1

**Published:** 2026-03-31

**Authors:** Ayk Waldow, Andreas Brachner, Nicolas Perriere, Winfried Neuhaus, Jörg Piontek

**Affiliations:** 1 Clinical Physiology/Nutritional Medicine, Department of Gastroenterology, Rheumatology and Infectious Diseases, Charité – Universitätsmedizin Berlin, 1 Hindenburgdamm 30, 12203 Berlin, Germany; 2 Competence Unit Molecular Diagnostics, Center for Health and Bioresources, AIT - Austrian Institute of Technology GmbH, Vienna, Austria; 3 BrainPlotting SAS, Institut du Cerveau et de la Moelle épinière, Paris, France; 4 Faculty of Medicine and Dentistry, Danube Private University, Krems, Austria

**Keywords:** Nanoscopy, STED, Tight junction, Claudin, Blood brain barrier, Endothelia, Epithelia, In vitro models

## Abstract

**Background:**

The composition of tight junctions (TJs) in epithelia is well understood, whereas that one in endothelial cells at the blood-brain barrier (BBB) is controversial. Although freeze fracture EM remains the gold standard for assessing TJ nano-structures, advances in fluorescence microscopy, such as stimulated emission depletion (STED) together with image analysis tools, allow immunostaining-based quantitative analysis of the protein composition and the nano-organization of TJ strands.

**Methods:**

Here, we present two approaches for quantitative analysis of the BBB TJ meshwork utilizing confocal and STED imaging together with open access image analysis tools CellProfiler, iLastik and ImageJ. For the first use case, a BBB model based on brain capillary endothelial-like cells (BCELCs) differentiated from human induced pluripotent stem cells (hiPSC) was employed. For the second one, monolayers of human primary brain microvascular endothelial cells (pBMVECs) were used.

**Results:**

STED analysis of BCELC monolayers revealed that here claudin-5 co-polymerizes with claudin-4 and − 6 into continuous TJ strand meshworks. Treatments with established TJ openers (1.4 M mannitol or *C. perfringens* enterotoxin-based claudin binders) strongly decreased transendothelial resistance (TEER) accompanied by a reduction in the nanoscopic co-localization of claudin-5/-4, -5/-6 and amount of junctional claudin-5. These findings suggested that in hiPSC-based BBB models multiple claudins together constitute TJs, leading to a tight paracellular barrier against solutes. Using STED imaging of pBMVECs immunostained for Cldn5, extensive meshworks of continuous TJ strands were detected. In contrast to the hiPSC-based model, other claudins were not detected. TEER and morphometric analyses of Cldn5-positive strands revealed that the barrier defect caused by claudin-5 binders is due to impaired TJ structural integrity.

**Conclusions:**

The findings suggest that Cldn5 is sufficient to form the tight paracellular barrier at the BBB. The presence of claudin-4, -5 and − 6 in hiPSC-derived cells reflects a mixed phenotype depending on their differentiation process. In sum, we demonstrated that, unlike confocal imaging, STED enables monitoring the composition and structural integrity of BBB TJ strands.

**Supplementary Information:**

The online version contains supplementary material available at 10.1186/s12987-026-00799-1.

## Background

**Fig. 1:**
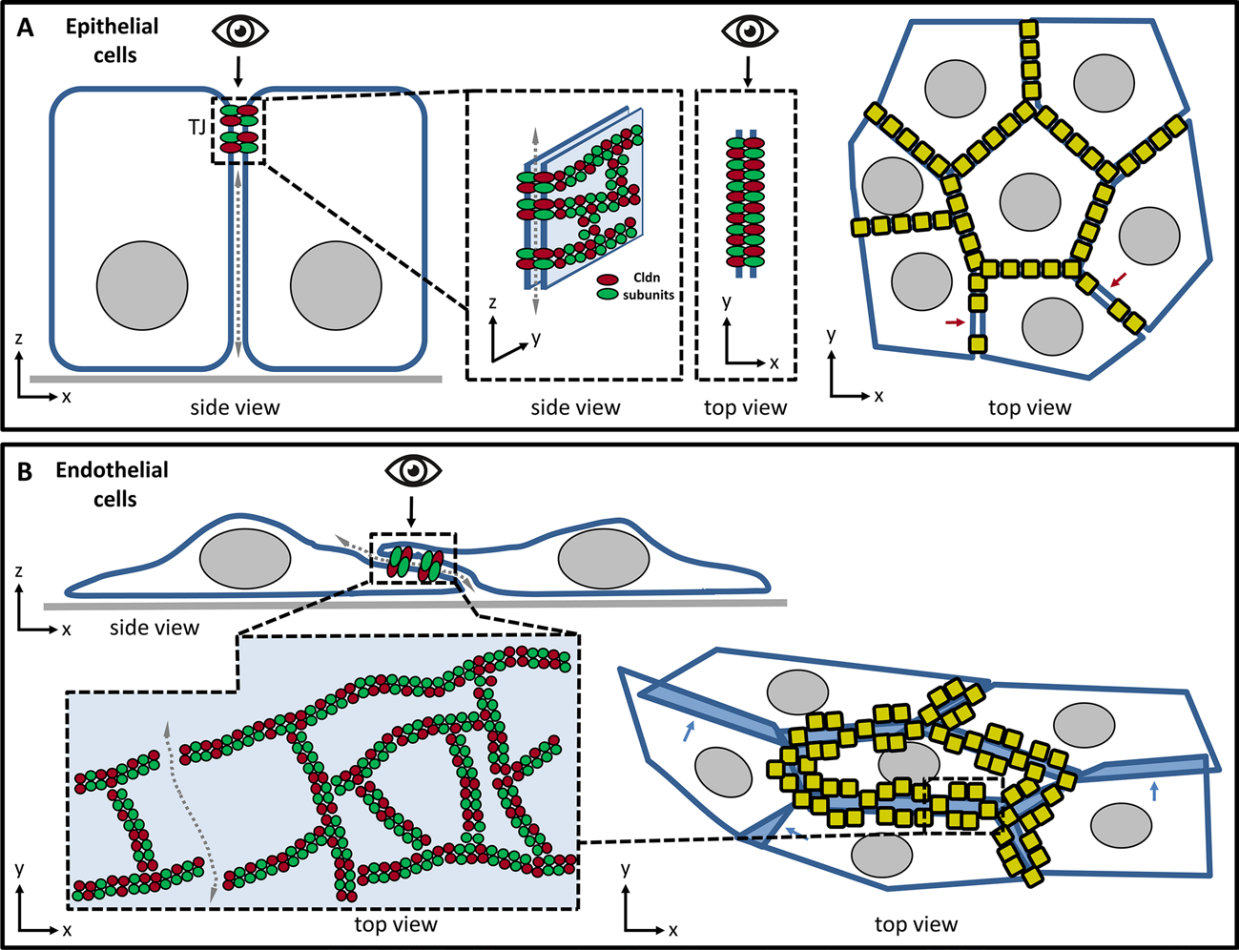
Schematic drawing depicting the position, orientation and elongation of TJs in epithelia and endothelia. (**A**) In epithelia, TJs are located at the most apical region of the lateral plasma membrane between adjacent cells (left, dashed box). Cell membrane (blue), paracellular permeation path (dashed arrow), nuclei (gray circles) and basement membrane (gray line) are shown. The backbone of TJ strands consists of claudin polymers with subunits (green and red ovals) interacting in cis (within one membrane) and in trans (between membranes). Meshworks of branched strands spread around the cells and along the apicolateral membrane (z-axis, middle, side view). When observed by light microscopy (eye symbol), the TJs are observed from the top (or bottom), which hinders to resolve the strand meshwork optically (top view, middle and right). In the top view (right), for simplicity, TJs are depicted as junctional bricks (yellow) containing multiple claudin subunits instead of showing individual subunits. The TJs form a continuous belt around the cells to occlude the paracellular cleft (shown for the middle cell). Discontinuities (red arrows) in the TJ belt cause paracellular leakage. (**B**) In brain microvessels, endothelial cells grow much flatter than most epithelial cells, with extended cell-cell overlaps [33, 70], representing the border between the apical and basolateral membranes (top, dashed box). Similarly, for cultured brain endothelial cells, the meshwork of TJ strands is mostly spread within the x-y plane, favouring the resolution of the strand meshwork when observed microscopically from the top or bottom (eye symbol). (Bottom, left) Top view of close-up of cell-cell overlap (dashed boxes) shows the meshwork of mostly continuous strands with individual claudin subunits (green, red circles). If gaps/discontinuities in the strands are present, paracellular leakage is caused (dashed arrow). The membrane plane is depicted in light blue. (Bottom, right) Top view of cultured endothelial cell monolayer. Overlaps of cell membranes (cell-cell overlaps) are shown as blue areas, for clarity, in part without TJs (blue arrows) and in part with TJs depicted at junctional bricks (yellow) containing multiple claudin subunits. Whereas in top views of epithelial cells TJs appear as lines, they appear as meshworks in endothelial cells. Thus, in this study, meshworks of TJ strands were imaged in the xy plane at cell-cell overlaps in endothelial monolayer cultures

The biological barriers between different body compartments are mainly formed by epithelia and endothelia. The paracellular barrier is here formed by the most apical cell-cell junctions in the lateral membrane, the tight junctions (TJ). In epithelia, the TJ spread around the cells forming a continuous belt, resulting in an efficient paracellular barrier against molecules (Fig. 1A). In so-called tight epithelia, the TJ also acts as a barrier against small ions [1]. However, in most peripheral organ endothelia, the TJ belt is rather discontinuous, resulting in the leakage of solutes through the paracellular pathway [2, 3]. This supports the exchange of metabolites between small blood vessels/capillaries and the tissue. In contrast, in the endothelium of brain blood vessels, a continuous TJ belt forms one of the tightest paracellular barriers in the (human) body with a transendothelial electrical resistance (TEER) of 500 to 6000 Ω∙cm^2^ [4, 5]. Here, the TJs form the paracellular part of the blood-brain barrier (BBB) that prevents the entry of toxic and other unwanted substances, pathogens or cells into the brain parenchym [6].

The backbone of TJs is constituted by claudin polymers (TJ strands, Fig. 1A) that build the actual paracellular barrier [7–9]. The claudin family comprises in mammals ~27 members, that are expressed in a tissue-specific manner [1, 9, 10]. Functionally, they can be grouped into barrier-forming claudins that seal the TJs also for small ions and channel-forming claudins building size-and charge-selective ion channels within the TJ strands. In addition, claudins can be classified into classic and non-classic claudins based on sequence homology and self-assembly properties [1, 9]. Other transmembrane TJ proteins, such as occludin, tricellulin, junctional adhesion molecules (JAMs) and angulins contribute to and regulate the paracellular barrier. Of the many cytosolic TJ-associated proteins, the scaffolding protein ZO1 is of particular importance since it regulates the TJ strand and TJ belt formation intracellularly by direct interaction with the transmembrane TJ proteins and liquid-liquid phase separation [11].

Epithelial TJs are much better understood than the endothelial BBB TJs. This is partly due to established in vitro models for epithelia [1, 12, 13]. In addition, for most epithelial tissues it is well characterized which claudins are expressed and part of the respective TJs [1, 14, 15]. In contrast, the claudin expression in BBB endothelia is less clear, although it is well established that Cldn5 is essential for sealing the BBB for small molecules [16]. Whether additional claudins significantly contribute to the barrier, and if so, which ones, is still controversial. Some studies emphasized an all-dominant role of Clnd5 [17–23] (https://cellxgene.cziscience.com/), while others reported a contribution of other claudins (Cldn1: [21, 24, 25], Cldn3: [26, 27], Cldn4: [28, 29], Cldn11: [13, 21, 30, 31], Cldn12: [16], others: [13, 30, 31]). This discrepancy may be partly due to different contexts (developing, mature or diseased BBB) but also largely due to methodological differences and limitations (bulk or scRNAseq, Western blot, Immunostaining, mass-spectrometry, antibody specificities).

To address this, we followed the idea that specificity of the claudin detection could arise from a better focus on the proteins within the functional relevant structure: The TJ strands. As the gold standard, TJ strands are detected by freeze fracture EM based on their unique ultrastructure [32–34], and freeze-fracture replica immunolabeling can be used to identify particular claudin subtypes within the strands [35]. However, the latter is strongly limited due to low labeling efficiency and unspecific background signals. An alternative is provided by super-resolution fluorescence microscopy. In this regard, Stimulated Emission Depletion (STED) microscopy was successfully used to resolve individual TJ strands and to identify claudin subtypes within strands reconstituted in non-polar cells or within endogenous strands of epithelial tissue [36]. For the BBB-relevant Cldn5, reconstituted TJ strand meshworks were previously resolved by STED and single molecule localization microscopy, SMLM [36, 37].

This study used STED microscopy to detect claudins in previously established in vitro models of the BBB: Brain capillary endothelial-like cells (BCELCs) differentiated from human induced pluripotent stem cells (hiPSC [38, 39]), human primary brain microvascular endothelial cells (pBMVECs) [40], and the mouse brain endothelial cell line cerebEND [12, 41, 42]. We were able to resolve claudin-positive TJ strands by STED microscopy in all models. However, the results differed, accounting for the varying differentiation state of the diverse models. The results support the idea that Cldn5 is the main and most critical constituent of BBB TJs and that Cldn5 is sufficient to form a continuous and distinct paracellular barrier at the BBB. The methodology of STED nanoscopy and quantitative colocalization and morphometric analysis reported in this manuscript can be used to further disclose composition, assembly, regulation and other mechanistic aspects of the BBB TJs.

## Materials and methods

### Cell culture

#### SBAD0201 cell culture and differentiation into BCELCs

The human induced pluripotent stem cell (hiPSC) line SBAD0201 had been generated by IMI StemBANCC, and was kindly provided by Dr Zameel Cader, University of Oxford. SBAD0201 cells were maintained on Matrigel^®^ (#BDL354230, BD Biosciences) coated 6-well plates (#140675, Nunc, Thermo Scientific) in mTeSR™1 medium (#100–0276, STEMCELL™ Technologies) in an incubator at 37° C, 95% humidity and a 5% CO2 containing atmosphere. Cultures were split twice a week 1:10 by incubation with a 0.5 mM EDTA/PBS solution and disaggregating the hiPSC colonies into smaller cell clumps (< 10 cells) by pipetting. Cells were seeded in mTeSR™1 medium containing 10 µM of the Rock kinase inhibitor Y-27,632 (#1254, Tocris). Differentiation was performed following the protocol established by Lippmann et al. [43] with minor modifications. Briefly, cells were detached with Accutase^®^ solution (#A6964, Sigma-Aldrich) and seeded with a defined cell density of 7500 cells/cm2 on Matrigel^®^ coated 6-well plates. Medium was changed after 24 h, and on the following day, the medium was changed to unconditioned medium (UM) to induce neuro-endothelial co-differentiation (UM; DMEM/F-12 (#21331020, Gibco™), 20% KnockOut™ Serum Replacement (# 10828028, Gibco™), 1% MEM NEAA (#11140035, Gibco™), 1 mM L-Glutamine (#G7513, Sigma-Aldrich), 0.1 mM β-Mercaptoethanol (#M3148, Sigma-Aldrich)). Differentiation in UM medium was done for 6 days with daily medium exchange, followed by 2 days in human endothelial-SFM medium (#11540386, Gibco™) supplemented with 0.5x B-27™ (#17504044, Gibco™), 20 ng/mL hbFGF (#F0291, Sigma-Aldrich) and 10 µM all-trans retinoic acid (#R2625, Sigma-Aldrich) (EC++). Cells were then seeded in EC + + medium at a density of 10^6^ cells/cm^2^ on 24-well ThinCert^®^ cell culture membrane inserts with 0.4 μm pores (#662641, Greiner Bio-One), coated overnight at 37 °C with a mixture of 400 µg/mL collagen IV and 100 µg/mL fibronectin (#C5533 and #F1141, both from Sigma-Aldrich). After 24 h, medium was exchanged for EC medium (without hbFGF and RA), and incubated for another day (d10). Starting on d10, experimental treatments were performed.

#### cerebEND cells

Mouse cerebEND cells were a kind gift from Prof. Carola Foerster and described in 2006 for the first time (Silwedel 2006). CerebENDs were cultivated on 0.5% gelatine (#22151, Serva)-coated flasks, in high glucose DMEM (#D5796, Sigma-Aldrich) supplemented with 10% fetal calf serum (#F9665, Sigma-Aldrich), 100U penicillin and 100 mg/mL streptomycin (#P4333, Sigma-Aldrich), in an incubator at 37° C, 95% humidity and a 5% CO_2_ containing atmosphere as described before [12]. Cells were routinely split 1:3, once a week. For transwell experiments, cells were seeded at a density of 4 × 10^4^ cells/cm^2^ on collagen IV coated 24-well ThinCerts^®^ with 0.4 μm pores ((#662641, Greiner Bio-One) and cultivated for 7 days, medium was changed three times per week. From day 7 to day 12 cells were treated with 100 nM hydrocortisone (#H2270, Sigma-Aldrich) added to the apical compartment with every medium change. Experiments were conducted on day 13.

#### Human primary brain microvascular endothelial cells (pBMVECs)

All human samples were obtained from BrainPlotting (iPEPS, Institut du Cerveau et de la Moelle épinière, Hôpital Universitaire de la Pitié-Salpêtrière, Paris, France), and harvested under the authorization number (CODECOH DC-2014-2229) in partnership with Sainte-Anne Hospital, Paris (neurosurgeon Dr. Johan Pallud). Patients gave their written informed consent. Human brain capillaries were isolated from cortex resections obtained from patients during tumor scheduled resection surgery diagnosed with glioma (grade II to grade IV i.e. glioblastoma). Enzymatic isolation of human brain capillaries was adapted from previously described protocols in the rat [44, 45]. Briefly, human brain samples were cleaned from meninges and excess of blood. Tissues were digested using an enzymatic mix and capillaries retained on a 10 µM mesh. The human microvessels obtained were treated with puromycin to positively select brain endothelial cells with the BBB phenotype (expressing P-gp) and subsequently cultured in EBM-2 (Lonza) to originate human primary brain microvascular endothelial cells (pBMVECs) grown in monolayers on Transwell^®^ inserts (Corning) for 8 to 12 days.

### TEER measurements

Transendothelial electrical resistance (TEER) was determined with an EVOM3 (Epithelial Volt/Ohm meter 3) device (World Precision Instruments, Sarasota, FL, USA) and STX2 chopstick electrodes (Merck Millipore) as described previously [46]. The initial TEER (0 h) was determined after equilibrating electrodes and cell cultures in fresh medium for 40 min at room temperature. Following the indicated time of treatment, TEER was reassessed. TEER values, given as Ω·cm², were calculated by subtracting the mean resistance value (Ω) of three blank (cell-free) membranes and multiplication with the membrane surface area (0.336 cm^2^).

### Expression and purification of GST-cCPE fusion proteins (cCPE)

GST fusion proteins of mutants of the claudin-binding domain (194–319, cCPE) from the *Clostridium perfringens* enterotoxin with known affinities to different claudins have been previously used to target claudins for TJ modulation in different in vitro tissue barrier models [12, 47–50]. The corresponding plasmids, expression and purification of GST-cCPE-S305P/S307R/S313H (GST-cCPE-SSS) binding to a broad spectrum of claudins including Cldn1-9, CPE-Y306W/S313H (cCPE-YWSH) with binding affinity shifted towards Cldn5 and GST-cCPE-Y306A/L315A (GST-cCPE-YL) as non-binding control were reported previously [12, 47–49, 51].

### Fixation, staining, antibodies

Prior to fixation cells were rinsed with PBS(+ Ca²^+^/+Mg^2+^) and then fixed with pre-cooled (-20 °C) 96% ethanol incubated for 10 min at -20 °C. Following fixation cells were rinsed with PBS(-/-) once for 5 min and afterwards blocked with blocking solution (1% (w/v) BSA, 2% (v/v) Goat serum, 0.05% Tween-20 in PBS (-/-)) for one hour. Staining was then performed with primary antibodies diluted 1:100 in blocking solution at 4 °C overnight. Primary antibodies used for staining were mouse anti-ZO1 (Invitrogen Thermo Fisher Scientific Inc., #39-9100), rabbit anti-Cldn4 (Invitrogen Thermo Fisher Scientific Inc., #36-4800), rabbit anti-Cldn5 (Invitrogen Thermo Fisher Scientific Inc., #34-1600), mouse anti-Cldn5 (Invitrogen Thermo Fisher Scientific Inc., #35-2500) and rabbit anti-Cldn6 (BiCell Scientific, #00206) for BCELCs. For pBMVECs Cldn5 was visualized with rabbit anti Cldn5 (Invitrogen Thermo Fisher Scientific Inc., #34-1600). After primary antibody staining the cells were rinsed with blocking solution for five times and afterwards incubated with the according secondary antibodies anti-mouse STAR ORANGE (Abberior, STORANGE-1001-500UG), anti-rabbit STAR ORANGE (abberior, STORANGE-1002-500UG), anti-mouse STAR RED (abberior, STRED-1001-500UG) and anti-rabbit STAR RED (abberior, STRED-1002-500UG) at 1:250 dilution in blocking solution at room temperature for one hour. After secondary antibody staining cells were rinsed twice with blocking solution and twice with distilled water for 5 min each and then mounted in Abberior solid antifade mounting media (abberior, MM-2013-2 × 15ML) on #1.5 H glass coverslips. After at least 24 h of hardening the samples were ready for imaging.

### Confocal and time-gated STED imaging

Confocal and STED imaging was performed on an abberior FACILITY LINE system (abberior, Germany) with an Olympus IX83 inverted microscope, Olympus UPLXAPO 60X (NA 1.42) objective and Olympus IMMOIL-F30CC immersion oil (refractive index of 1.515). For detection avalanche photodiodes were used allowing detection of single photon counts. For image acquisition the software LiGTHBOX (abberior) was used. Multi-color confocal and STED images were obtained in line-mode with a pixel size set to 100 nm per pixel for confocal overview images and 20 nm per pixel for close-up STED images. For all images Cldn5 was detected via the STAR RED dye excited with a pulsed 640 nm laser and detection at 650–755 nm, while other markers were excited with a pulsed 594 nm laser detected via the STAR ORANGE dye at 571–630 nm. For depletion a pulsed 775 nm STED laser was used. Time-gated detection was set from 0.75 to 8.75 ns. Pixel dwell time was set to 5 µs and a line averaging of at least 5 times was used. Prior to imaging the system was automatically aligned with nanoparticles (abberior autoalignment sample) and afterwards manually fine tuned to ensure optimal settings for STED imaging. Regions of interest (ROIs) for STED imaging were selected based on the enrichment of one or two of the following junctional proteins at cell-cell contacts in confocal scans: Cldn4, -5, -6, ZO1, occludin and VE-cadherin. For occludin and VE-cadherin, STED images were not acquired or analyzed since the photon count were too low for further analysis. To estimate the minimally detected width of TJ strands, STED images were post-processed with the Abberior LiGTHBOX software (version 2025.10.22547) using first the filter Image/Enhance/Denoise and afterwards the filter Deconvolve/Intensity, both with default settings.

### Image analysis - image segmentation

For segmentation two different approaches were used based on the follow-up applications. In case of colocalization analysis, a simple segmentation creating a mask from the confocal images by either manually drawing the mask or using a two-class Otsu thresholding method followed by application of a Gaussian blur with a radius of 5 pixels. These masks were then used to limit colocalization analysis of STED images to junctional areas within the images. Segmentation for co-occurence and meshwork measurements were done with assisted machine learning (iLastik [52]). Part of the pre-processed STED images with enhanced contrast and subtracted background were used as training data. Segmentation was achieved by a two class (background and tight junctions) pixel classification based on a Random Forest classifier generating a probability map which was ultimately used to create a binary mask of the tight junction. For parameters smoothed pixel intensities, edge filters and texture descriptors with sigma values ranging from 0.3 to 3.5 were chosen. Annotations for both classes were added throughout the training set manually while the model was trained simultaneously. Once the segmentation appeared sufficient the complete test set was segmented, then revisited and new annotations were added to improve the model. After multiple reiterations of training, images for analysis were pre-processed and then segmented by the trained model for subsequent meshwork analysis.

### Image analysis - colocalisation

For colocalisation measurements in RepSox treated samples, manually cropped STED images were used for determining Pearson correlation coefficient using the coloc2 plugin in imageJ [53]. For mannitol and cCPE treated conditions images were cropped with masks from the confocal images and then the Pearson correlation coefficient was determined for the STED images in CellProfiler [54]. Co-occurence was determined by calculating the overlap between segmented STED images of both channels derived from the assisted machine learning segmentation.

### Image analysis - meshwork analysis

For analyzing TJ meshworks we utilized the segmented STED images obtained from the assisted machine learning approach and analyzed them regarding meshwork complexity and degree of fragmentation. For determining the degree of fragmentation we determined the amount of individual objects over the total area of the meshwork both obtained via imageJ. The quotient obtained this way measures how fractured a TJ meshwork is with increasing count of objects leading to an overall increase in fragmentation, however in order to obtain a measure for the continuity of a meshwork we used the reciprocal value of fragmentation, hence leading to total area over object count which coincides with the *mean object size*. In practice this was obtained by using the *analyze particle* function in imageJ in order to determine the size of each object. For analyzing descriptors of the TJ meshwork segmented STED images were used to determine the average size of the meshes. Individual branches, junctions and shape descriptors of meshes were measured in skeletonized images of the segmented STED images. Altogether, we determined mesh area, mesh count, mean branch length, branch count, junction counts, tortuosity, circularity and quotients derived from these measures.

### Image analysis – intensity measurements

To assess claudin expression, intensity was measured from raw STED images cropped for the TJ area by using the masks derived from confocal images described above. Since the avalanche photodiodes detect and count the total number of photons in each pixel for the set acquisition time frame, bigger masks lead to higher photon counts. Therefore, the photon count was normalized to the size of the confocal mask used for the segmentation resulting in a photon count over area measure for intensities.

### Data analysis and statistics

Measurements obtained in either imageJ or CellProfiler were subsequently analyzed in either GraphPad Prism 7 (Graphpad Software) or RStudio (R Core Team, 2024, https://www.R-project.org). In GraphPad Prism 7 the data was first tested for normal distribution by multiple common tests such as Shapiro-Wilk, D’agostino & Pearson test and Anderson-Darling test and from that a likelihood of normality with p < = 0.05 was determined. For normal distributed data, Student’s t-test was used to determine significances, in case of non-normal distributed data, Mann-Whitney test was used. In case of log-normal distributed data prior to testing with Mann-Whitney test it was logarithmized. Due to only single comparisons in these cases no multiple comparison correction method was applied. Prior to performing statistical tests in R, the data was tested for distribution by using the Shapiro-Wilk test. In case of normal distributed data the Levene-test was performed to test for equal variance and then followed up by the Welch-test for non-equal variance data or Student’s t-test for equal variance data with subsequent usage of the Bonferroni-Holm method to account for multiple comparisons. In case of non-normal distributed data the Kruskal-Willis test was performed to test for significant differences among the data followed up by Dunn-test for pairwise comparison with Bonferroni-Holm correction to account for multiple comparisons. For non-normal distributed data sets without multiple comparisons Wilcoxon test was performed for each pairwise comparison.

### Generation of schematic claudin hetero-polymer model

The model generation was based on the hypothesis that the different classic claudins (including Cldn4, -5, -6, -10b and others) assemble into polymeric strands with a similar overall architecture [9, 55–59]. Similarly as conducted previously [60], multiple copies of a claudin oligomer structure (8IBli model) obtained by all-atom molecular dynamics (MD) simulations of membrane-embedded Cldn10b homo-dodecamers [55] were aligned using PyMOL (Version 3.1.4.1, Schrödinger, LLC, Germany) to build a claudin polymer containing 40 claudin subunits. This polymer consisted of ten neighboring and interlocked tetrameric scaffold units. This Cldn10b homo-polymeric structure was used as a rigid body template for generation of schematic claudin hetero-polymers by assigning, either Cldn4, Cldn5 or Cldn6 to one of the 40 subunits. To screen different potential *cis*- and *trans*-interfaces, the different claudins were placed at varying subunit positions using these polymer models and multiple sequence alignments of human and mouse classic claudins (Cldn1-10, -14, -15, -17, -19; Clustal WS, Jalview version 2.11.1.0, Waterhouse [61]. The residues at the resulting subunit interfaces were compared with the interfaces in the existing all-atom models for Cldn10a, -10b and − 15 homo-oligomers [55, 56] and schematic Cldn1, -2, -3 homo- and hetero-oligomers [60]. The most plausible hetero-interfaces (for instance regarding matching potential electrostatic interactions) were selected for the final hetero-polymer for visualization with PyMOL.

## Results

### Brain capillary endothelial-like cells (BCELCs) derived from SBAD0201 cells as a model for analysis of TJ organization

The majority of in vitro BBB models only partially reproduce the robust paracellular barrier that is present in vivo [62–64]. Furthermore, species differences may limit the transferability to the human BBB. To overcome the limited accessibility of human primary brain endothelial cells, human induced pluripotent stem cells (hiPSCs) were previously differentiated into brain capillary endothelial-like cells (BCELCs [38]). These cells were used to develop 2D monolayer, 3D spheroid and microfluidic BBB models [62, 65, 66]. In this study, we focused on 2D monolayer models, which are more applicable to high-resolution imaging. We employed a recently developed BCELC model, based on the hiPSC line SBAD0201, which exhibits very high transendothelial electrical resistance (TEER, up to 6000 Ω·cm²), indicative of a highly restrictive barrier, and substantial expression of BBB markers, including Glut-1, VE-Cadherin, and Claudin-5 [39]. We utilized these BCELCs monolayers **(i)** as a BBB model expressing multiple claudins (Cldn1, -3, -4, -6, -7, -11 mRNA [39]), **(ii)** to establish multi-color STED analysis of TJs in an BBB model and **(iii)** to analyze if other claudins than Cldn5 contribute to the paracellular barrier of this model.

### SBAD0201-derived BCELC monolayers form a robust and manipulable solute barrier

**Fig. 2:**
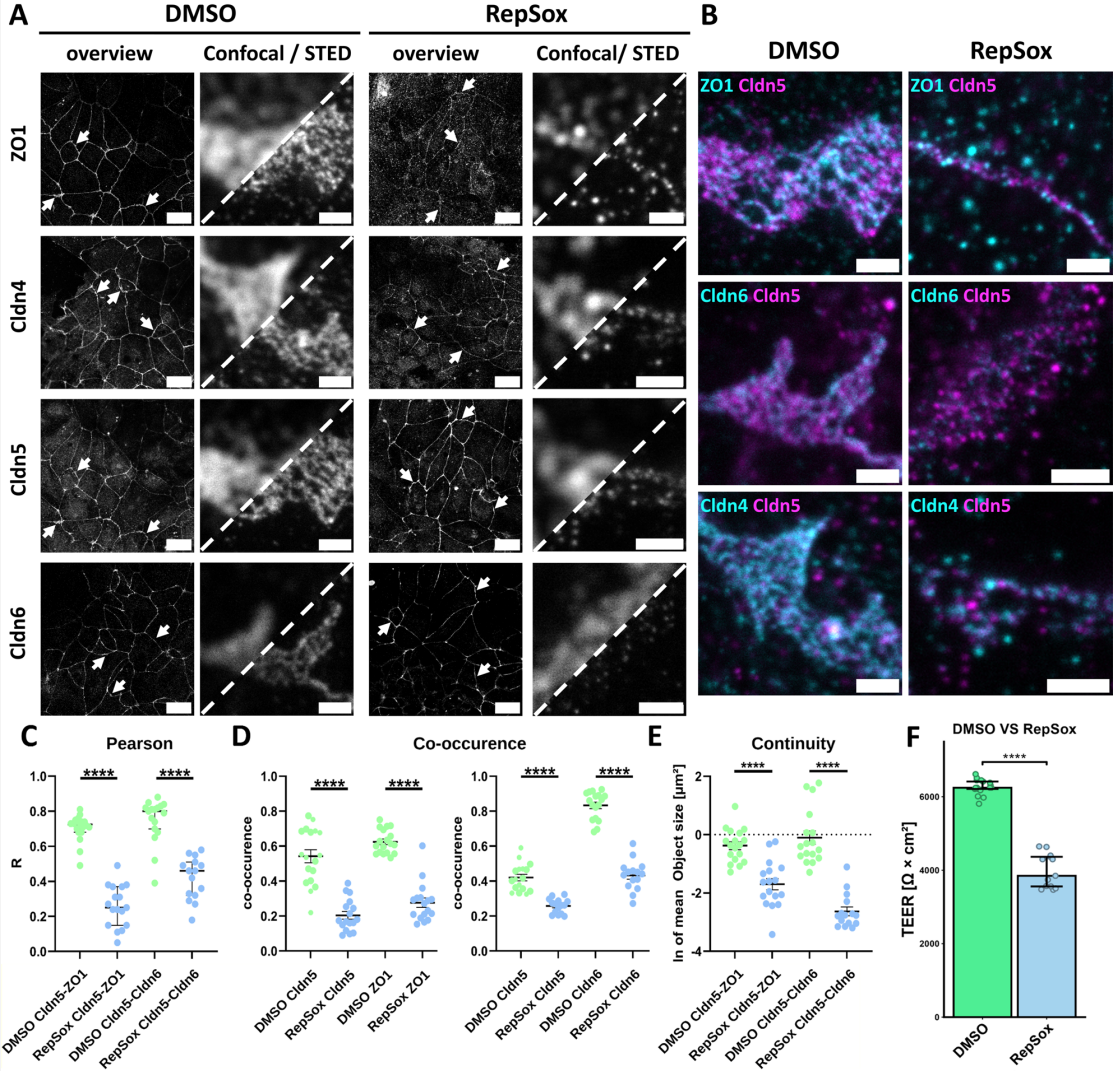
RepSox effects on Claudin nanoscale organization in BCELCs visualized by STED. Immunostaining (with rabbit/mouse primary and STARred/STARorange-conjugated secondary antibodies) of SBAD0201 cells differentiated to BCELCs via two protocols differing in the final step of differentiation (24 h DMSO or RepSox). (**A**-**B**) Overviews and STED images showing expression and junctional localization of respective TJ proteins in DMSO and RepSox culture conditions. (**A**) Confocal overviews and split confocal/STED close-ups of junctional regions (arrows) for ZO1, Cldn4, Cldn5, Cldn6. For each protein an example junctional region of interest (ROI) is shown. For the DMSO condition, meshworks of TJ strands were resolved by STED but not confocal microscopy. (**B**) Representative merged STED images for DMSO and RepSox conditions showing co-integration into common TJ strand meshworks for Cldn4, Cldn5, Cldn6 and ZO1. Incomplete colocalization indicates at least partial segregation of the proteins within the meshwork. RepSox reinforced the segregation and led to fragmentation of the meshwork. Scale bars in overviews are 10 μm, in STED images 1 μm. (**C**-**E**) Quantitative image analysis. (**C**) Pearson correlation coefficient R and (**D**) co-occurence measured as the overlap between both TJ proteins (Manders coefficient) exemplary for Cldn5/ZO1 and Cldn5/Cldn6 as measure for colocalization between TJ proteins. RepSox reduced all of these colocalization parameters. Median with IQR, *n* ≥ 15 images. (**E**) Continuity determined as mean objects size (inverse of fragmentation) exemplary for merged meshworks of Cldn5/ZO1 and Cldn5/Cldn6 to assess meshwork integrity. RepSox reduced continuity of the meshworks. Median with IQR, *n* ≥ 15 images. (**F**) TEER [Ω∙cm²] measured for both culture condition before fixation. Median with IQR. *n* = 17 filters for DMSO and *n* = 13 filters for RepSox condition from 2 independent experiments. ****p < 0.0001 determined by Mann-Whitney test

First, the effects of different differentiation conditions on forming a paracellular solute/electrolyte barrier were compared. In particular, we tested the compound RepSox, a well-known selective inhibitor of the TGF-β type 1 receptor (TGFβRI) which was previously shown to modulate TJ and barrier formation in other BBB models, including hiPSC/BCELCs [67–69]. We compared the effect of RepSox on TEER after 10 days of cultivation in the presence and absence of retinoic acid (RA). The culture conditions containing RA showed a very high median TEER of 6247 Ω∙cm², while the additional presence of RepSox from day 9 to day 10 during the differentiation significantly decreased TEER down to a median of 3848 Ω∙cm². The absence of RA led to a very low average TEER of either 128 Ω∙cm² for RepSox or 133 Ω∙cm² for the DMSO control (Fig. 2F, Fig. S1A, Table S1). The results verified that RA supported the formation of a strong paracellular solute barrier and showed that the presence of RepSox in the current setting weakened the formation of this barrier in SBAD0201-derived BCELCs. Nevertheless, in the presence of RA and Repsox, a considerable barrier was still formed, since the corresponding TEER was still much higher than the values for the conditions without RA. Thus, the two differentiation conditions (RA ± RepSox) produced proper barriers of different strengths for further analysis.

### Establishment of multi-color STED analysis of TJs in the BCELCs BBB model

We hypothesized that the aforementioned barrier difference was related to a different nanostructure of TJs and that STED microscopy could resolve this in endothelial monolayers, in which the meshwork of TJ strands spread along the observation/focal xy plane (Fig. 1). Thus, the differently cultured BCELCs served as a model system for establishing a multicolor STED analysis of the barrier-determining TJ organization.

Initially, immunostainings against several claudins (Cldn1, -3, -4, -6, -7, -11) that were detected in these BCELCs at the RNA level [39] were analyzed by confocal and STED microscopy. Then, we focused on the barrier-forming claudins Cldn4, -5 and -6 [71–74], which were clearly enriched in the junctional area of BCELCs (Fig. 2A). The junctions were detected via the TJ-associated protein ZO1. The advantage of STED became apparent by comparing the confocal with the STED images: While the confocal signals were blurred, the STED signals provided higher resolution and revealed smaller substructures that were not separable in confocal images (Fig. 2A, split images). In the STED images of BCELCs differentiated without RepSox (strongest barrier), the meshwork of TJ strands, and in many cases also individual TJ strands and meshes got visible. This was the case for Cldn4, -5 and − 6 as well as for the associated ZO1 (Fig. 2A, DMSO). Partly, the strands appeared doted and not completely continuous. This could be due to gaps in the strands, incomplete detection e.g. because of steric hinderance or partial segregation of the different claudins. Interestingly, merge of two channels showed that (i) the Cldn5 strand meshwork was clearly associated with a ZO1 meshwork, (ii) Cldn4, -5 and − 6 were integrated into common meshworks, and (iii) the claudins did not colocalize homogeneously but segregated partly within the meshworks. Consequently, the strands appeared more continuous/complete in the merge than in the single channels (Fig. 2B, DMSO).

For the two different conditions (± RepSox), no clear differences in the meshworks of TJ strands were evident at the confocal level. In contrast, the STED images showed that the overall appearance of the TJ strand meshwork changed from a rather continuous meshwork with small gaps (< 100 nm) likely occupied by other claudins (without RepSox) to a more open dotted pattern (with RepSox) with bigger gaps (> 200 nm) (Fig. 2A). When comparing the merged STED images of Cldn5 and Cldn4 or Cldn6, this effect was observed more clearly. While Cldn5/Cldn6 and Cldn5/Cldn4 together form a continuous meshwork of claudin strands in the condition without RepSox, no continuous meshwork was visible for the RepSox condition (Fig. 2B). In general, the TJs were defined “discontinuous” when the merged images of the claudin channels frequently showed gaps > 100 nm (> five pixels, ea 20 nm) in multiple strands of the meshwork. TJs without such gaps were defined “continuous”.

These findings were quantified by determining the degree of colocalization for Cldn5/ZO1 and Cldn5/Cldn6 by calculating the Pearson correlation coefficients and the signal co-occurrence as well as by determining the signal continuity in the merged images. RepSox significantly reduced all parameters: Pearson coefficients and co-occurences for Cldn5/ZO1 and Cldn5/Cldn6 as well as continuity of Cldn5/ZO1 and Cldn5/Cldn6 meshworks (Fig. 2C-E).

The results showed that STED imaging enabled the detection of meshworks of TJ strands and their claudin composition. The meshwork integrity (as evaluated by quantification of meshwork continuity and colocalization of TJ proteins (pearson & co-occurence), Fig. 2A-E) correlated with the barrier function of the TJs (TEER, Fig. 2F). The nanoscopic localization made it possible to assign the immunoreactive signals to TJ strands, the subcellular structure in which claudins execute their barrier function. This improved the discrimination between non-specific and specific antigens recognised by the antibodies, which is a common issue with claudin antibodies [26, 75]. Overall, the data indicate, that in BCELCs, Cldn5 associated with ZO1 and formed mixed TJ strands at least together with Cldn4 and Cldn6 to form a functional paracellular solute barrier.

### Hyperosmolarity and claudin targeting diminish the BCELC barrier

**Fig. 3:**
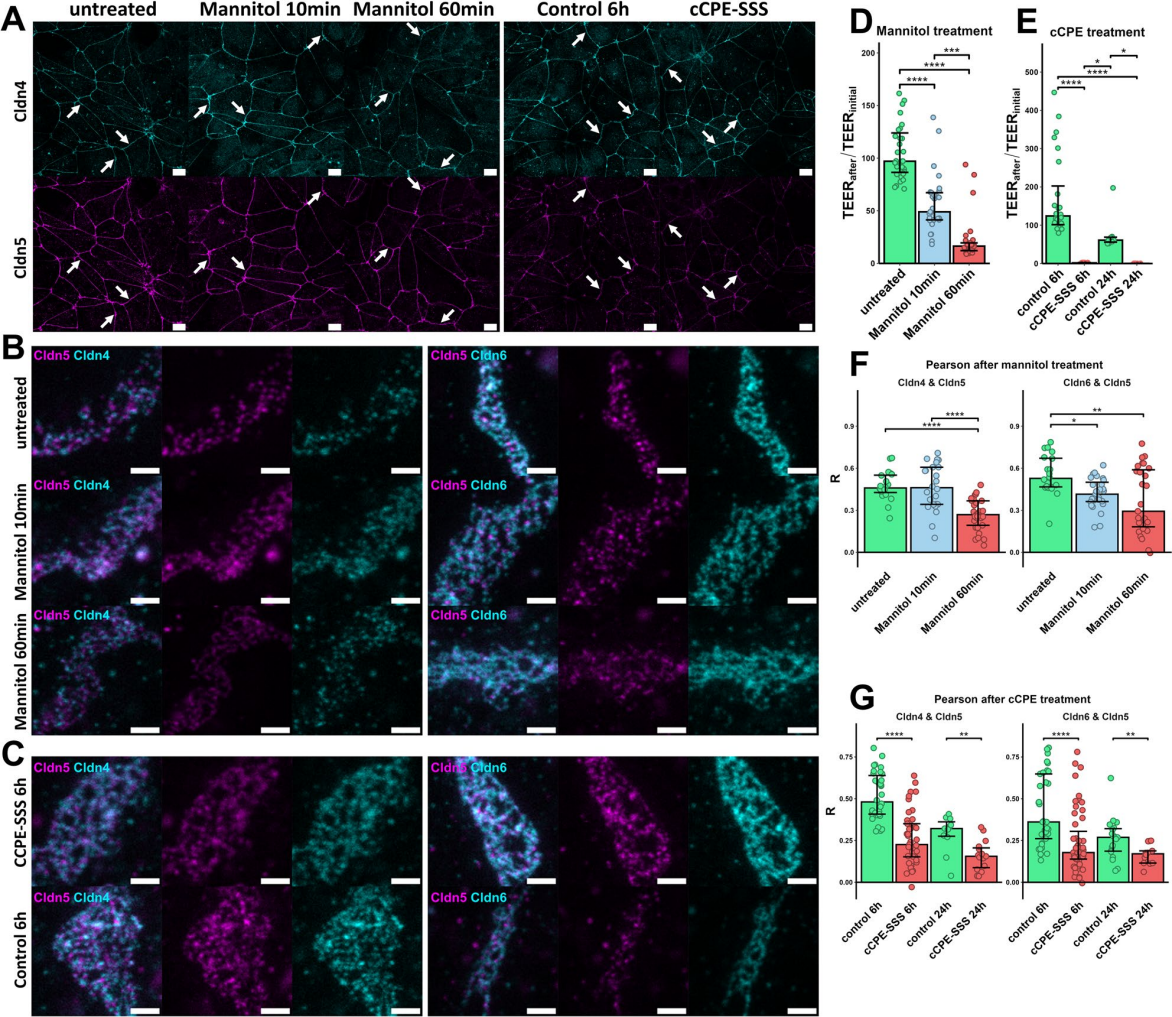
Effect of mannitol and cCPE treatment on the claudin nanoscale organization in BCELCs. Qualitative and quantitative analysis of BCELC monolayers on filter inserts untreated, incubated on both sides for 10–60 min with 1.4 M mannitol, on both sides for 6–24 h with 50 µg/ml cCPE-SSS (broad-spectrum claudin binder) or respective PBS controls, fixed and stained against claudins. (**A**) Representative confocal overviews of Cldn4/Cldn5 co-immunostainings for untreated, mannitol, PBS controls 6 h and cCPE-SSS treatments. Clear junctional signals (arrows) were obtained for the untreated, control conditions and 10 min mannitol. For 60 min mannitol and cCPE-SSS treatment, the junctional signals for Cldn4 and Cldn5 were – if at all – only slightly diminished. Scale bars, 10 μm. (**B**,** C**) Representative STED images (merge and single channels) of junctional regions of interest (ROIs) for the different conditions and Cldn5/Cldn4 and Cldn5/Cldn6 co-stainings. Both claudin pairs were co-detected in common strand meshworks with partial colocalization. Scale bars, 1 μm. (**D**,** E**) Relative TEER values after treatment for 10–60 min with 1.4 M mannitol (**D**) or for 6–24 h with cCPE-SSS (**E**) demonstrating the respective impairment of the paracellular electrolyte barrier. Median with IQR. For (**D**) *n* ≥ 29, for (**E**) at 6 h incubations *n =* 24 and for 24 h incubations *n* ≥ 6. (**F**,** G**) Colocalization measured by determining the Pearson correlation coefficient R for Cldn4/Cldn5 and Cldn6/Cldn5 after 10 min and 60 min 1.4 M mannitol treatments (**F**) or 6 h and 24 h cCPE-SSS treatments (**G**) in comparison to the respective control (untreated/PBS). The mannitol and cCPE treatments reduced the colocalization. Median with IQR. For (F) *n* ≥ 20, for (**G**) at 6 h incubations *n* ≥ *33* and for 24 h incubations *n* ≥ 11. Each *n* represents an individual STED image with a claudin meshwork analyzed. Adjusted P-values were determined with Kruskal-Wallis test followed by post-hoc Dunn’s-Test with multiple comparison adjusted by using Holm-method. In (**G**) Wilcoxon test was performed for comparisons of cCPE conditions vs. their respective controls. *p < 0.05; **p < 0.005; ***p < 0.0005; ****p < 0.0001

To further test whether the nanoscopic TJ organization resolved by STED imaging reflects the barrier properties of the BCELC monolayers, a condition known to weaken the paracellular barrier formed by TJs, hyperosmolarity induced by treatment with 1.4 M mannitol [76, 77], was applied. First, the effect of this treatment on TEER of the BCELC monolayers was tested. Mannitol treatment led to a drastic decrease in TEER with a 50% decrease after 10 min and 90% after 60 min, resembling a profound breakdown of the solute barrier (Fig. 3D). In addition, to specifically target claudins, we used a mutant of the claudin-binding domain of the *Clostridiumperfringense*nterotoxin (*cCPE*-S305P/S307R/S313H, cCPE-SSS), which binds to a broad spectrum of classic claudins, including Cldn1-9 [47–49, 51]. It was shown that cCPE binding sequesters claudins blocking their proper integration into TJs that in turn weakens the paracellular barrier [9, 12, 47, 48, 51, 78]. Consequently, treatment for six or 24 h with cCPE-SSS reduced TEER close to blank filter values (Fig. 3E). Thus, both the non-specific mannitol and the claudin-directed cCPE treatment clearly compromised the paracellular solute barrier in this BBB model.

### Hyperosmolarity-mediated barrier opening is associated with alteration of junctional claudins

Subsequently, the differentially treated BCELC cultures were immunostained and the TJ strand meshworks analyzed by confocal & STED microscopy. In confocal overview images of the monolayers, no clear effect of the hyperosmolarity treatments was visible. Similar to the controls, Cldn4, Cldn5 and Cldn6 were strongly enriched at cell-cell junctions after 10 min and 60 min of mannitol treatment (Fig. 3A, Fig. S1B). The high resolution STED images of junctional regions showed for each condition a dot like-pattern for Cldn4, Cldn5 and Cldn6, all present in the same meshwork (Fig. 3B). Quantification of the STED signal intensities in the junctional region revealed a slight decrease for Cldn5 after 10 min and a strong reduction after 60 min of mannitol treatment (Fig. S1C). For Cldn6, a reduction was observed after 10 and 60 min of mannitol treatment, whereas for ZO1 no reduction was detected. However, Cldn4 showed a strong increase after 10 min of mannitol treatment, which did not persist after 60 min (Fig. S1C). In addition, Cldn5 colocalization with Cldn4 was reduced after 60 min mannitol treatment, and that with Cldn6 was even reduced after 10 and 60 min of hyperosmolarity (Fig. 3F). The data indicated that (i) mannitol induced changes in the TJ nano-organization within 10 min, (ii) it reduced the amount of claudin-5 and the interconnection of claudins in TJ strand meshworks within 60 min and (iii) this contributed to the hyperosmolarity-induced TJ opening.

### cCPE-mediated barrier opening is also associated with alteration of junctional claudins

Similar to hyperosmolarity, cCPE treatment did not result in a clear effect at the level of confocal overviews. As with the controls, Cldn4, Cldn5, and Cldn6 were strongly enriched at cell-cell junctions in BCELC monolayers after incubation with cCPE-SSS (Fig. 3A, **F**ig. S1B). STED images of the junctional regions showed a dot-like pattern for Cldn4, Cldn5, and Cldn6 under all conditions (Fig. 3B). However, the Cldn5 and Cldn6 intensities in junctional regions imaged with STED were clearly reduced after 6 h of cCPE-SSS treatment as compared to controls (Fig. S1C). Furthermore, Cldn5 colocalization with Cldn4 and with Cldn6 was prominently weaker after 6 h and 24 h cCPE-SSS treatment compared to the respective controls (Fig. 3G).

In sum, the data obtained under TJ-modulating conditions showed that, unlike confocal microscopy, STED analysis can detect alterations in TJ nano-organization that determine the paracellular barrier properties of the BCELC BBB model.

### Nanoscopic analysis of TJ organization in an immortalized mouse brain endothelial cell line

In addition to the human hiPSC-based BBB model, we also intended to use a model based on a brain endothelial cell line with high Cldn5 expression and of a different species. For this, we employed the immortalized murine cerebellar microvascular endothelial cell line cerebEND [42]. We verified our previous finding [12] that these cells form a paracellular solute barrier that can be compromised by treatment with cCPE variants (Fig. S2L). Cldn5 and ZO1 could be detected by immunostainings, and STED analysis showed that ZO1-associated meshworks of TJ strands can also be resolved in these brain endothelial cells that predominantly express Cldn5 (Fig. S2A-K). In addition, we detected that cCPE treatment reduced the continuity of TJ strands in this system (Fig. S2M), which went along with weakening of the barrier (Fig. S2L). However, the cerebEND cells showed a strongly heterogeneous appearance of the Cldn5 and ZO-1 signals with respect to their expansion, continuity, density of strand and mesh-like structures and enrichment in the periphery of the cells. This limited more detailed analyses, including robust morphometry of TJ nano-organization.

### Nanoscopic analysis of TJ organization in human primary brain microvascular endothelial cells (pBMVECs)

Given the fact that hiPSC-derived BCELCs most likely represent brain endothelial cells in an intermediate or not fully differentiated state [79], human primary brain endothelial cells with a close-to physiological TJ expression pattern and morphology, should be analyzed. In order to achieve this aim, transwell cultures of pBMVECs, freshly isolated and cultivated by BrainPlotting SAS, were analyzed [40, 80].

### Cldn5 junctional strand meshworks of pBMVECs can be resolved by STED nanoscopy

**Fig. 4:**
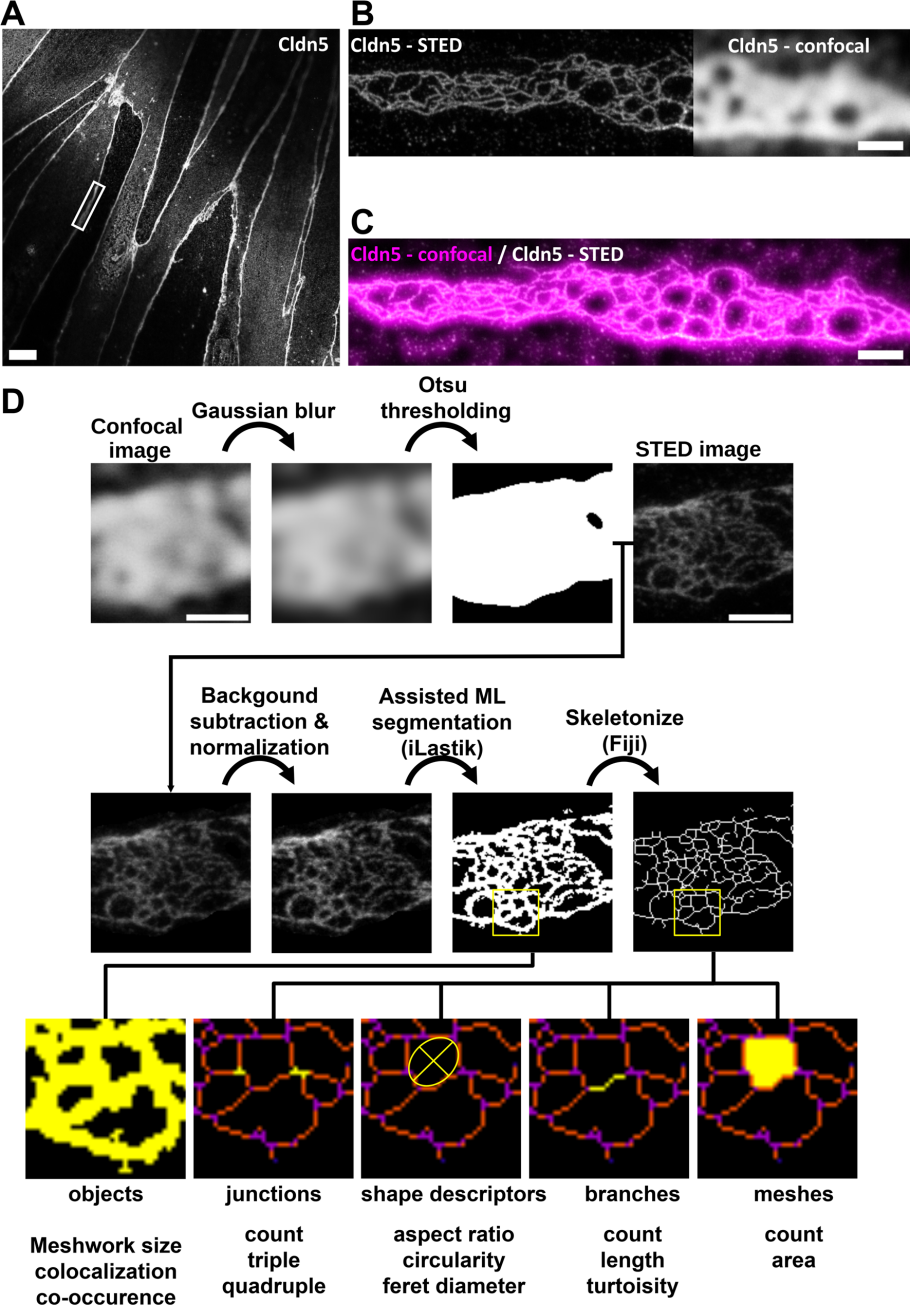
STED image analysis of TJ organization in human primary brain microvascular endothelial cells (pBMVECs) cultured on Transwell^®^ inserts. Cells were stained for Cldn5. (**A**) Representative confocal overview of pBMVEC monolayers showing typical elongated cell morphology with strong junctional Cldn5 signal. White box indicates exemplary junctional region of interest (ROI) for STED imaging. Scale bar, 10 μm (**B**,** C**) Close ups of the ROI indicated by the box in (**A**) for side by side comparison (**B**) and overlay (**C**) of Cldn5 confocal and STED images highlighting the substantial increase in resolution for subsequent image analysis. Meshworks of Cldn5-positive TJs can be resolved by STED microscopy but only insufficiently by confocal imaging. At this resolution (~ 50–100 nm), individual continuous and branched Cldn5-positive TJ strands can be detected. Scale bars, 1 μm. (**D**) Scheme for quantification of STED images for Cldn5 meshworks obtained from pBMVEC demonstrated on an example image. Preprocessing in Fiji included masking by use of the blurred and thresholded confocal image obtained with the Otsu thresholding algorithm followed by, background subtraction and intensity normalization of the STED image. Preprocessed images were segmented with the assisted machine learning software iLastik and then converted into a skeleton image in Fiji. Quantifications were performed from either segmented binary images or skeletonized images and included the mentioned parameters. Scale bar, 1 μm

The pBMVEC monolayers grown on Transwell^®^ filters were stained for two claudins that were reported to be expressed in brain endothelial cells: Cldn5 [16] and Cldn11 [30, 31]. While no clear junctional signals were obtained for Cldn11 (Fig. S3), robust signals were distinctly enriched at cell-cell junctions and were clearly detected for Cldn5 by confocal microscopy (Fig. 4A).

Strikingly, corresponding STED images of cell-cell contacts revealed meshworks of continuous Cldn5-positive TJ strands that could be distinctly detected (Fig. 4B, C). This was aided by the flat cell morphology of pBMVECs, resulting in junctional cell-cell overlaps (Fig. 1B). Most of the elongated junctional strand meshworks resembled the complex meshwork of highly branched TJ strands, as detected by freeze-fracture EM of brain capillary samples and BBB in vitro models [12, 26, 38]. In particular, they resembled TJ strand meshworks detected in BCELC monolayers and BBB 3D spheroids derived from SBAD0201 cells [65]. In the STED images, the mesh diameters ranged between ~ 125 and ~ 1000 nm. The Cldn5-positive linear structures were detected with an average width of 118 ± 26 nm (full width at half maximum, FWHM, mean + SD), and widths down to *≤* 90 nm (25% percentile) for raw data and *≤* 55 nm (25% percentile) after denoising and deconvolution (see methods). The strand width detected by freeze-fracture EM is ~ 10 nm. Thus, as expected this resolution was not reached by STED light microscopy. Nevertheless, it was sufficient to assign the linear Cldn5-positive structures in many cases to individual TJ strands.

In addition to the elongated junctional meshworks of continuous Cldn5 strands, other arrangements of Cldn5 strands were also detected. In fact, the Cldn5-positive structures showed a strong heterogeneity. They ranged from (i) areas with homogeneous strand densities, (ii) areas with very different strand densities, (iii) areas with clear discontinuities/breaks in the strands presumably reflecting paracellular leaks, (iv) strand meshworks that were not connected to cell-cell contact presumably reflecting junctional patches that were demolished due to cytoskeletal/mechanical/tension-dependent forces, to (v) potentially internalized strands (Fig. S4). However, these detected structures that do not fit to canonical junctional elements have to be further characterized in future studies, for instance using 2D- or 3D-STED and respective marker costainings. Nevertheless, the 2D-STED used here, but not confocal imaging, clearly resolved all of these Cldn5 substructures.

In order to investigate differences in the nanoscopic structure of the TJ strand meshworks, a procedure for quantitative image analysis was established. The workflow is depicted in Fig. 4D. It contains the steps pre-processing with Fiji, segmentation utilizing assisted machine learning via iLastik, quantification with Fiji and data processing with R. For each condition a training set of at least 40 images was collected, while the test set to perform the analysis consisted of at least 10 images per condition. Quantifications to analyze different parameters of the meshworks e.g. branch length, number of junctions, total length, total area, mesh size and others were performed.

### cCPE-mediated TJ opening in pBMVEC monolayers is reflected by alteration of TJ nano-organization

**Fig. 5:**
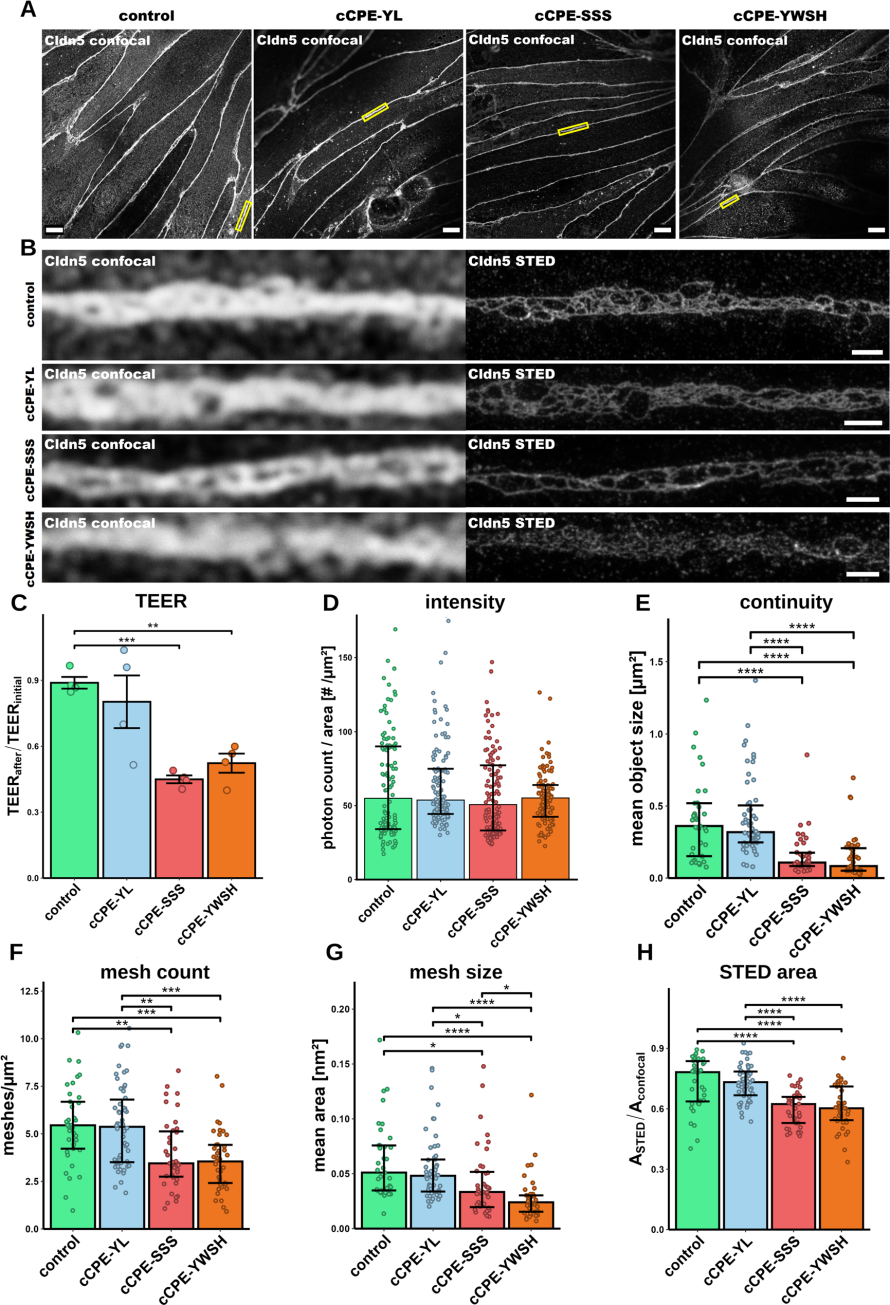
cCPE treatment affects nanoscale organization of Cldn5 in pBMVEC. (**A**) Representative confocal overviews of Cldn5 immunostainings of pBMVECs cultured on Transwell inserts, untreated (control) or treated on both sides for 6 h with 25 µg/ml cCPE-YL (non-binding control), cCPE-SSS or cCPE-YWSH (Cldn5 binders). Prominent presence of Cldn5 at cell junctions was detected for all conditions. Boxes highlight junctional regions of interest (ROIs) chosen for STED imaging. Scale bars, 10 μm. (**B**) Confocal and STED close-ups of junctional Cldn5 in the boxes indicated in (**A**). Confocal images could not reveal clear differences between the conditions. In contrast, STED imaging revealed a more fragmented and/or less complex strand meshwork pattern for cells treated with cCPE-YWSH or cCPE-SSS, compared to controls. Scale bars, 1 μm. (**C**) TEER values as ratio after/before treatment. cCPE-SSS and cCPE-YWSH reduced TEER clearly. *n* ≥ 5, each *n* represents an individual filter from two independent experiments. (**D**-**H**) Morphometric quantification of the Cldn5 STED images. (**D**) Intensity (photon count/area) measured in Cldn5 STED images. No difference between the different conditions was detected. Median with IQR and individual values. Each *n* represents an individual STED image from 4 different filters with *n* ≥ 108 for each condition. (**E**) Continuity of Cldn5 strand meshworks after treatment to measure meshwork integrity, given as mean object size (see methods). cCPE-SSS/-YWSH reduced the continuity. Each *n* corresponds to the mean object size of meshworks within a single STED image with *n* ≥ 37 for each condition. (**F**) Mesh count normalized to Cldn5 positive area of the confocal image to recognize changes in TJ meshwork complexity. cCPE-SSS/-YWSH reduced mesh count indicating decrease in a meshwork complexity. Each *n* represents a single STED image with *n* ≥ 37 for each condition. (**G**) Average size of complete meshes in Cldn5 meshworks. cCPE-SSS/-YWSH reduced mesh size. Each *n* represents a single STED image with *n* ≥ 37 for each condition. (**H**) Ratio of STED area per confocal area to determine extent of Cldn5 presence at TJ after treatment. Each *n* represents an individual STED image with *n* ≥ 37 for each condition. For (**C**) mean ± SEM, for (**D**-**H**) median with IQR and individual values. For (**C**) adjusted p-values were determined by unpaired Welch test and for (**D**-**H**) by Kruskal-Wallis test with post-hoc Dunn’s test. Holm-method was used in both cases to adjust for multiple comparison. *p < 0.05; **p < 0.005; ***p < 0.0005; ****p < 0.0001

Next, we intended to test whether the nanoscopic organization of Cldn5-based TJ strands, as resolved by STED imaging, reflects the barrier properties of the pBMVEC monolayers. In order to compromise the TJ barrier, we incubated the cells with Cldn5-binding cCPE variants: cCPE-S305P/S307R/S313H (cCPE-SSS) – the broad spectrum claudin binder, cCPE-Y306W/S313H (cCPE-YWSH) – binding affinity shifted towards Cldn5 and cCPE-Y306A/L315A (cCPE-YL) – not-binding control. Before treatment, the TEER of the pBMVEC monolayers was 531 ± 111 Ω∙cm² (mean ± SD), reflecting a well-developed endothelial barrier [40, 45]. cCPE-SSS treatment reduced TEER of the pBMVEC monolayers (Fig. 5C), concordant with the effects previously seen in BCELCs (Fig. 3). cCPE-YWSH reduced TEER to a similar extent as cCPE-SSS whereas cCPE-YL control did not have an effect (Fig. 5C). Thus, cCPE variants weaken the barrier of pBMVEC monolayers in a claudin-dependent manner.

Then, the pBMVEC monolayers were stained for Cldn5, imaged, and a morphometric analysis was performed to investigate the effect of cCPE on TJ nano-organization. On the confocal level, no clear effect of the treatment with any of the cCPE variants was detected (Fig. 5A). On the contrary, STED images showed much more discontinuities in the strand meshwork for cCPE-YWSH-treated compared to untreated and cCPE-YL-treated pBMVECs (Fig. 5B). For cCPE-SSS the meshwork appeared to be also affected, however, this was less clear. Quantification revealed that the Cldn5 intensity in the junctional area was not affected by the cCPE treatments (Fig. 5D). Nevertheless, compared to the controls, treatment with cCPE-YWSH or cCPE–SSS decreased (i) the *mean object size* (Fig. 5E) and (ii) the *Cldn5-STED-positive area normalized to the Cldn5-confocal positive area* (Fig. 5H). Both parameters are measures for the meshwork continuity (see methods, meshwork analysis). Thus, the data indicated that cCPE treatment increased the fragmentation (decreased continuity) of the strand meshworks. Moreover, the complexity of the meshwork was impeded as indicated by a reduced amount of complete meshes (Fig. 5F). Furthermore, the size of the detected meshes was reduced (Fig. 5G), likely reflecting a less efficient detection of large meshes after segmentation caused by the strand fragmentation. Overall, fragmentation and reduced mesh count, in particular, clearly demonstrated the impairment of the TJ integrity by cCPE-SSS/-YWSH. Thus, the data indicated that cCPE binding to Cldn5 decreases the complexity and continuity of TJ strand meshworks, resulting in increased solute permeability.

## Discussion

This study showed that STED microscopy of immunostained BBB in vitro models enables monitoring of protein composition and structural integrity of brain endothelial TJ strands. The findings suggest that in hiPSC-based BBB models, TJ strands are formed by multiple claudins, resulting in a very tight paracellular barrier against solutes. On the other hand, in pBMVECs Cldn5 was verified to be the all-dominant constituent of TJ strands. Respective strand meshworks could be resolved by STED and analyzed by segmentation-based morphometric analysis. It was shown that barrier opening by hyperosmolarity or claudin targeting was related to a disturbance in the integrity of Cldn5-based TJ strands.

The claudin composition of the TJs of BBB endothelia is still disputed. Several studies reported the expression of multiple claudins in different BBB models [13, 21, 24–31, 65, 81], while others reported predominant expression of Cldn5 [17, 19, 20, 22, 23]. A very recent proteomic study reported that Cldn5 is by far the most abundant claudin expressed in human and mouse brain microvessels [82], and only minor amounts of Cldn1, Cldn7 and Cldn11 could be detected. However, in human pBMVEC cultures large amounts of Cldn11 were detected. Similarly, in total brain samples Cldn11 was the most abundant claudin [82], most likely due to its strong expression in the abundant oligodendrocytes [83]. This raises the question, whether the Cldn11 expression detected in human pBMVEC cultures is the result of a contamination with oligodendrocytes, a consequence of dedifferentiation in vitro or an BBB-relevant expression? In our study, no clear junctional Cldn11 was detected in human pBMVEC cultures. In contrast, Cldn5 was clearly detected in TJs of the pBMVEC monolayers. This fits to the expression data in brain microvessels [82] and other data on the RNA expression in brain endothelial cells [18, 19]. On the other hand, the current study detected Cldn4, Cldn5, and Cldn6 in the TJs of BCELCs. This aligns with the detection of various claudins at the RNA level in BCELCs derived from SBAD0201 cells and other hiPSC lines [39, 65, 84]. However, the difference between primary and hiPSC-derived BBB models suggests that the presence of Cldn4, -5 and − 6 in BCELCs reflects a mixed phenotype depending on the differentiation process of these model cells. This is a relevant finding since hiPSC-derived models are increasingly used. Advantages of BCELCs include the expression of several endothelial markers, including BBB-relevant transporters, as well as BBB-like very low paracellular solute permeability. In addition, unlike human primary cells, the BCELCs were not exposed to pathological conditions, such as a tumor environment, that could potentially affect their phenotype. However, this does not ensure that the composition, structure, and dynamics of the BCELC TJ, and therefore the regulation of paracellular permeability, reflect those of the mature human BBB. This should be considered when further optimizing BCELC differentiation protocols [39, 65, 67–69, 84, 85].

Importantly, the essential function of Cldn5 at the BBB is demonstrated by a BBB leakage to solutes < 800 Da in knockout mice, resulting in death shortly after birth [16], transient BBB opening after transient Cldn5 knockdown in mice, reduced Cldn5 expression in numerous disorders including schizophrenia, depression, traumatic brain injury and epilepsy as well as by pathogenic missense mutations causing alternating hemiplegia of childhood [22, 69].

Cldn5 can form TJ strands autonomously [35–37, 86]. It strengthens the barrier against small ions and other solutes when overexpressed in epithelial or endothelial cells that also express other claudins endogenously [72, 87]. However, it was unclear whether Cldn5 expression alone is sufficient to form an efficient solute barrier as has been shown for other strand-forming claudins (Cldn1 to -4, -7, -10, -15, -19 [36, 74, 88]. On the one hand, self-assembly of Cldn5 into a barrier was indicated by sequence similarity with those claudins [9, 74, 89]. On the other hand, strands reconstituted by Cldn5 expression in unpolar cells appear in freeze fracture (FF) replica differently than the continuous strands formed by other claudins: Spaced intramembranous particles just on one fracture face (E- but not P-face), resulting in a particle coverage along a “strand” far less than 100% [86, 90, 91]. This raised the question of whether significant gaps leading to paracellular leakage might be present if Cldn5 is not supported by another TJ protein? Strikingly, it was demonstrated very recently that Cldn5 expression in Cldn-null epithelial cells leads to barrier formation [92]. In addition, Kashihara et al. showed an example FF-EM image with beaded intramembranous TJ particles on the E-face with only very few gaps. However, a detailed analysis of the E-/P-face distribution, gap sizes and abundance was not reported. Nevertheless, the data indicate a particle coverage close to 100% for these Cldn5-based barrier-forming strand, similar to the particle coverage at the mature BBB in vivo [93]. This is consistent with the concept that (i) the barrier is formed by a polymeric and thus continuous structure and (ii) that the presence of Cldn5 at the BBB could be sufficient for such a structure. However, it cannot be ruled out that other TJ components, for instance occludin play a significant role in TJ stand- and barrier formation, and that additional claudins contribute, at least under pathological conditions.

On the other hand, coexpression of Cldn5 with Cldn3 in unpolar cells increases the particle coverage along a strand, leading to an in vivo BBB-like TJ ultrastructure [33, 93, 94]. However, unlike earlier reports [26, 95], Cldn3 KO mice strongly suggest that Cldn3 is not present, or is at least not necessary for an intact BBB in mice [75]. Nevertheless, it is not known if this is also valid in humans and other reports still argue for a contribution of Cldn3 [27]. Furthermore, a dSTORM analysis of the nanoscale architecture of BBB TJs (bEND.3 cells, BCELCs, brain tissue sections and mouse brain capillaries) detected Cldn5 clusters spaced by gaps often > 400 nm [96], much larger than the gaps detected in other studies by freeze-fracture EM [86, 90, 92–94]. Importantly, the small gaps (10–50 nm) seen for BBB TJs in vivo on one fracture face are filled by particles in the complentary fracture face (particle coverage on P- and E-face together ~ 100%). The large gaps detected by dSTORM could correspond to large breaks (100–500 nm) in TJ strand meshworks as found for leaks in epithelia under pathological conditions [97] or in peripheral endothelia [2]. Thus, the nanostructure detected by dSTORM is not compatible with the idea that a continuous and tight solute barrier is constituted mainly by Cldn5. Consequently, Sasson et al. suggested that other TJ proteins occupy the gaps between the Cldn5 clusters and complete the barrier-forming structure. Alternatively, the detection of spaced clusters could be due to limited epitope accessibility for the antibodies, which could be caused by steric hindrance. Along this line, we often obtained punctate junctional claudin signals when establishing STED imaging for epithelial and endothelial TJs, while trying different fixations and antibodies. For instance, for the pBMVECs we found only conditions in which continuous signals were obtained either for Cldn5 or for ZO1 but not for both. Strikingly, meshworks of mostly continuous strands reconstituted in TJ-free cells were detected by single molecule localization microscopy (SMLM) when directly the fluorescence of YFP-Cldn5 or YFP-Cldn3 fusion proteins was used [37]. In addition, nanobodies against the YFP-tag or ligands to SNAP-tags were efficiently used to detect continuous strand formed by many different claudins using STED microscopy [36, 57]. In sum, the STED data for pBMVECs provided in the current study fitted to the freeze-fracture EM and SMLM images obtained previously for reconstituted Cldn5 strands [37, 86] and strongly support the concept that Cldn5 is the main constituent of protein polymers that form the paracellular diffusion barrier.

The striking advantages of STED nanoscopy over freeze-fracture EM are that (i) specific claudins are detected, (ii) claudins residing in both membranes of the cell-cell contact are detected, (iii) the complete junctional belt surrounding a cell can be imaged and not only just a small patch that is fractured by chance at the TJ level, (iv) colocalization studies with other proteins are possible. In principle, STED nanoscopy can also be performed in 3D and in combination with more markers, for instance membrane or cytoskeletal markers, to pinpoint the subcellular localization and orientation of the strand meshworks in the membrane plane in more detail. However, in this study, we avoided STED z-stacks (either with diffraction limited z-/axial resolution (2D-STED) or isotropic STED with super-resolution in all three dimensions (3D-STED)) to avoid photobleaching, weaker signal-to-noise ratios and lower xy-/lateral resolution. In addition, membrane labeling was hindered by the fixation conditions. These limitations could be addressed in future studies.

For pBMVECs, TEER and morphometric analysis of the Cldn5-postive strands revealed that the barrier defect caused by claudin-5 binders is strongly related to a disturbed structural integrity of TJs. Concerning TEER, similar results were previously obtained with cCPE-YWSH in other mammalian in vitro BBB models [12]. However, alteration of junctional Cldn5 could not be resolved by the use of standard confocal microscopy. In contrast, we showed here that STED imaging can overcome this limitation and reveals functionally relevant characteristics of the TJ nanostructure. Though, it should be mentioned that increased tracer permeation through the disintegrated strand meshwork could not yet be directly visualized.

So far, morphometric analysis of TJ strand meshworks detected by freeze fracture EM were largely based on their manual identification [97–101]. On the other hand, different (semi-automatic) segmentation approaches have been used for TJs detected at lower resolution by epifluorescence and confocal microscopy [57, 102–104] or for TJ strand meshworks detected by STED or SMLM [36, 37]. In the current study, we used point operations, filters, machine learning-based segmentation and automatization. This analysis workflow can be further improved given the constant progress in AI-based tools. In any case, detection of the TJ meshworks in a protein-specific and high-contrast manner is a major advantage over the manual or semi-automatic morphometric analysis of freeze fracture EM images. Thereby, the established methodology in this study opens the way for a more detailed analysis of the nanostructure of BBB TJs.

**Fig. 6:**
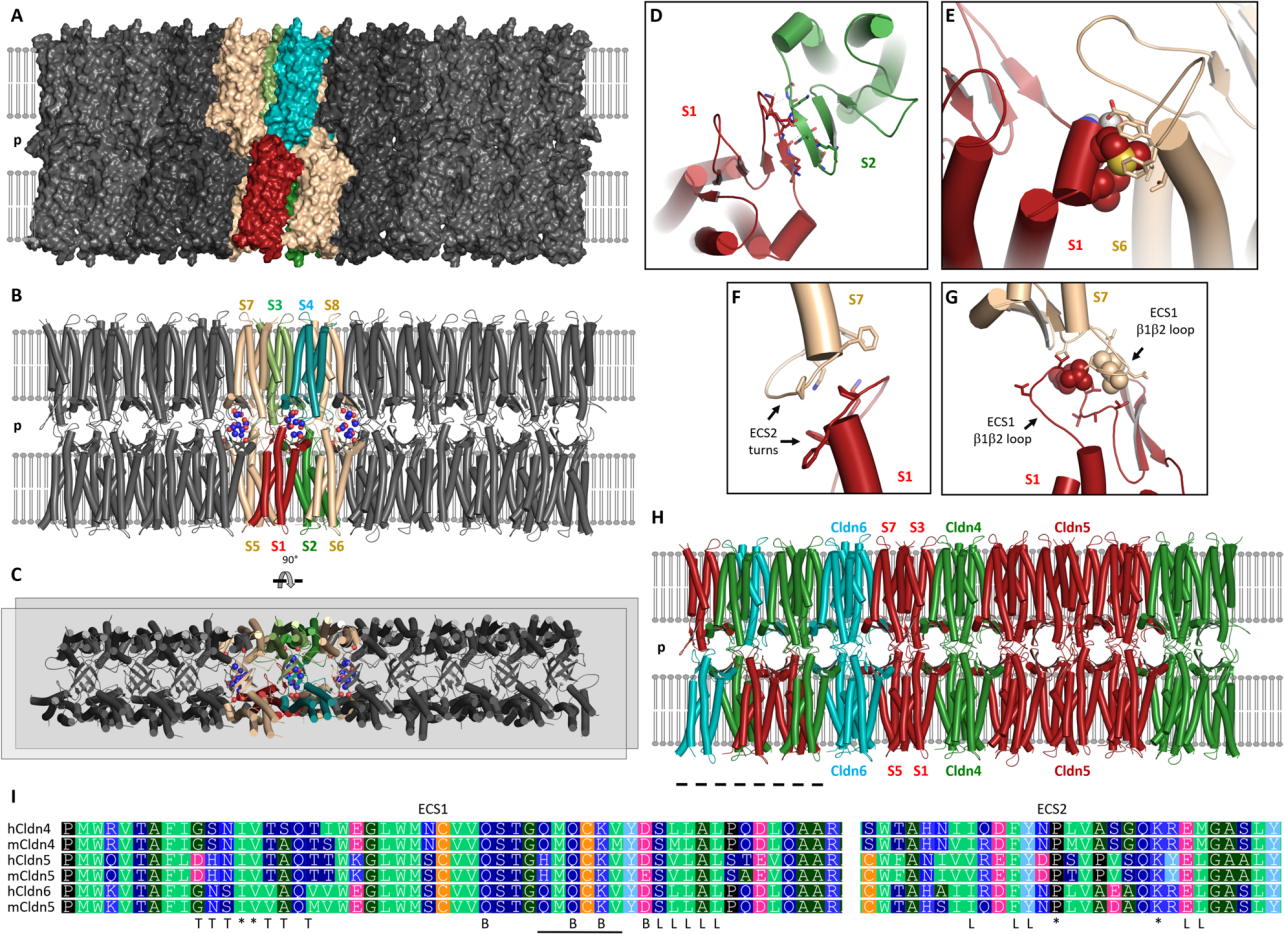
Schematic model of hetero-polymeric TJ strands formed by Cldn4, -5 and − 6 in BCELCs. Based on structural models generated for Cldn10a, -10b and Cldn3 [55–57] (**A-C**) Segment of claudin polymer at TJ. Multiple claudin subunits embedded in the two lipid bilayers are shown in different colors, each subunit corresponds to one oval in Fig. 1, the paracellular gap is labelled (p). (**A**) Surface representation of the claudins. (**B**) Cartoon representation of the protein secondary structure elements. Four subunits (S1-S4) constituting the tetrameric scaffold (pore in channel-forming claudins) are shown in red, green, dark green and cyan. These and four neighboring subunits (S5-S8, beige) form together the octameric unit (S1-S8) that is repeated along the claudin polymer. Residues (Q56, K65, D68) conserved among many barrier-forming claudins and proposed to contribute to electrolyte barrier formation are shown as sticks for three neighboring tetrameric scaffolds. Polar and charged atoms of these residues are shown as spheres. (**C**) View turned by 90° showing the anti-parallel double row of claudins in a membrane from the top. The two double rows from both cell membranes are joint via trans-interaction between the extracellular segments (ECS) of the claudins (A, B) resulting in the polymer (Joint double rows model [105]. (**D-G**) Classic claudins were proposed to share this strand architecture, since sequence motifs at key cis- and trans-interfaces are conserved [9]. (**D**) Face-to-face *cis*-interface between ECS1-β4 strands, e.g. of S1 and S2. Main chain H-bonds indicated as dashed lines and side chains supporting electrostatic *cis*-interactions shown as sticks. (**E**) Linear *cis*-interface between ECS1-ECH region of one subunit (e.g. S1, key residues as spheres) and ECS2 of another subunit (e.g. S6, key residues as sticks). (**F**) Trans-interface between two ECS2 turns (e.g. S1 and S7), key residues as sticks. (**G**) Trans-interface between two ECS1 β1β2-loops. Key conserved residues as spheres, key residues differing between claudins as sticks. (**H**) Proposed *cis*-&*trans*-match/compatibility between Cldn4, -5 and − 6 leading to hetero-polymers. The conservation of key residues at the face-to-face- and linear-cis- interfaces makes interactions between these claudins very conceivable. The ECS1 and ECS2 trans-interfacial residues are only partly conserved. Nevertheless, homo-tetramers of subunits at positions S1, S3, S5 and S7 would be free of any potential mismatch. Thus, such Cldn4, Cldn5, and Cldn6 tetramers could be joint by the mentioned common cis-interfaces. It is also conceivable that homo-oligomeric segments larger than tetramers are formed (example shown for Cldn5). In addition, it is not excluded that the sequence differences at the trans-interfaces do not (fully) prevent a corresponding interaction. This may lead to subunit arrangements shown as examples on the left side (dashed line) of the polymer. In total, sequence and oligomer model comparison are consistent with the formation of mixed TJ strands consisting of Cldn4, -5 and − 6. (**I**) Sequence alignment for human and mouse Cldn4, -5 and − 6; ECS1, ECS2 and adjacent regions (Clustal WS, Jalview). Note the high sequence homology that is also given at the residues/regions involved in linear- (L) and face-to-face (_) cis-interface, other key interfacial residues (*) and barrier former motif (B). Residues differing at the ECS1-β1β2 trans-interface are also labelled (T)

As mentioned above, the relevance of the contribution of Cldn4 and Cldn6 to the BBB in vivo is questionable. Nevertheless, the corresponding data obtained with BCELCs provide relevant mechanistic insight into the assembly of TJ strands, which is discussed below:

The structure of monomeric claudins is largely known whereas that of polymeric claudin strands is less clear. Models based on Cldn15 crystal packing, mutagenesis, molecular docking and molecular dynamics simulations suggest that TJ strands consist of joint double rows (JDRs) of claudins [9, 56, 98, 105, 106]. The models were mainly generated for Cldn15-like ion pore-containing homo-polymeric strands. Even less understood is how the strands that constitute a barrier for small electrolytes are formed. Different strand architectures (JDR-like and others) have been suggested for Cldn5 and other barrier-forming claudins [9, 57, 98, 107, 108]. So far, no atomistic models were generated for claudin hetero-polymeric strands. Although it is clear that in most tissues hetero-polymeric claudin strands are formed, at least for hetero-compatible claudins [7, 14, 36, 60, 94, 109], the determinants for hetero-(in)compatibility are largely unknown. Nevertheless, it was suggested that defined sequence motifs shared among the subgroup of classic claudins contribute to the ability for hetero-oligomerization [9, 36]. Importantly, Cldn4, -5 and − 6 share these motifs, while Cldn11 does not. Thus, these sequence patterns fit to the finding that Cldn4, -5 and − 6 are able to copolymerize. However, (i) at which oligomer positions they interact, (ii) in *cis* and/or in *trans*, (iii) within the octameric core building block of strands (Fig. 6B) or if homo-oligomers segregate from each other within hetero-polymeric strands is unclear [56, 60]. Nevertheless, the STED images of BCELCs revealed for the first time that Cldn5 can form common strand meshworks with other claudins in polar endothelial or epithelial cells. Alternatively, it was conceivable that Cldn5-containing meshworks and meshworks formed by other claudins largely exclude each other as found for Cldn3 and Cldn11 [36], Cldn3/-16/-19 and Cldn10b [110], and Cldn14 and Cldn6/-9 [111]. In order to understand claudin hetero-polymerization in general and for Cldn5 in more detail we generated a schematic model for Cldn4/-5/-6 hetero-polymers (Fig. 6) based on Cldn10 models [56, 60]. This model features the JDR architecture proposed for classic claudins with tetrameric and octameric building blocks. Cis- and trans-interfaces proposed to be compatible between Cldn4,-5 and − 6 but not with Cldn11 are indicated (Fig. 6).

We propose that combining (a) modeling of the strand structure and (b) STED-based determination of the ability of various claudins (and their mutants) to form hetero-polymeric strands can provide new insights into TJ assembly and, consequently, the regulation of paracellular permeability.

## Conclusion

The findings of this study suggest that hiPSC-based BBB models (BCELCs) exhibit a mixed stem cell/endothelial phenotype with TJ strands formed by multiple claudins including Cldn4, -5 and − 6, resulting in a very tight paracellular barrier against solutes. In contrast to this multi-claudin phenotype in BCELCs, we provide evidence that Cldn5 is the all-dominant component of TJ strands in pBMVECs. STED nanoscopy made it possible to resolve the respective strand networks. Subsequent analyses based on segmentation, morphometry, and quantitative colocalization analyses revealed that barrier opening by hyperosmolarity or claudin targeting is related to disturbances in the integrity of Cldn5-based TJ strands. This was not achievable with conventional confocal microscopy. Thus, the STED-based nanoscopic methodology reported here can be used in future studies to further disclose the composition and regulation of TJs at the BBB.

## Supplementary Information

Below is the link to the electronic supplementary material.

Supplementary Material 1

## Data Availability

All data generated or analyzed during this study are included in this published article, the supplementary information file or are available from the corresponding author on reasonable request.

## Abbreviations

BBB: Blood-brain barrier
BCELC: Brain capillary endothelial-like cells
cCPE: Claudin binding domain of Clostridium perfringens enterotoxin
Cldn: Claudin
hiPSC: Human induced pluripotent stem cells
pBMVEC: Human primary brain microvascular endothelial cells
SMLM: Single molecule localization microscopy
SSS: S305P/S307R/S313H
STED: Stimulated emission depletion microscopy
TEER: Transepithelial electrical resistance
TJ: Tight junctions
YL: Y306A/L315A
YWSH: Y306W/S313H
