# Supplementary material for: Nanoscopic analysis of tight junction organization in in vitro blood-brain barrier models
Source: Fluids Barriers CNS. 2026 Mar 31;23:68. doi: 10.1186/s12987-026-00799-1 (PMC13156884; doi:10.1186/s12987-026-00799-1)
Supplement: Supplementary file 1 — Supplementary Material 1 [file 12987_2026_799_MOESM1_ESM.pdf]

Supplemental Material

Nanoscopic analysis of tight junction organization in *in vitro* blood-brain barrier models

Ayk Waldow[1], Andreas Brachner[2], Nicolas Perriere[3], Winfried Neuhaus[2,4*], Jörg Piontek[1*]

[1]Clinical Physiology/Nutritional Medicine, Department of Gastroenterology, Rheumatology and Infectious Diseases, Charité – Universitätsmedizin Berlin, 1Hindenburgdamm 30, 12203 Berlin, Germany

[2]Competence Unit Molecular Diagnostics, Center for Health and Bioresources, AIT - Austrian Institute of Technology GmbH, Vienna, Austria

[3]BrainPlotting SAS, Institut du Cerveau et de la Moelle épinière, Paris, France.

[4]Faculty of Medicine and Dentistry, Danube Private University, Krems, Austria

*corresponding author

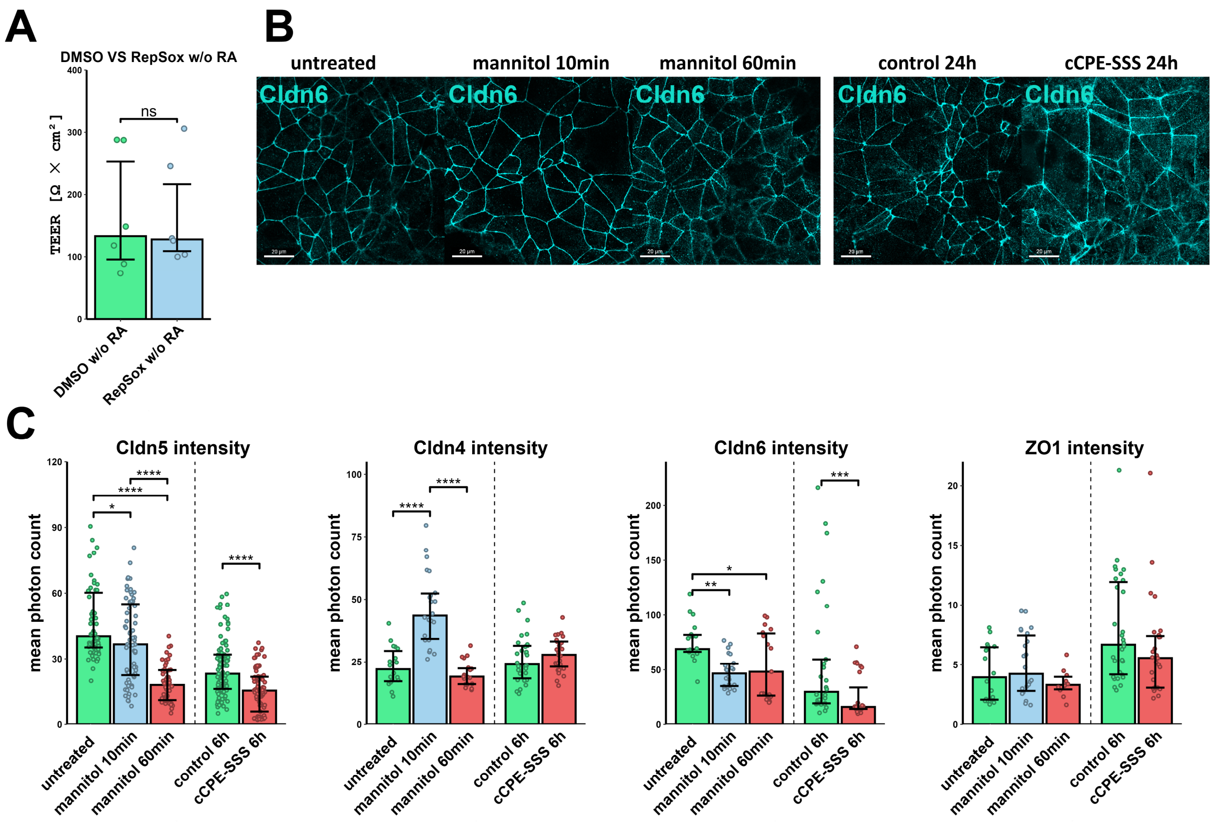

Figure S1 (A) Low TEER [Ω·cm²] for SBAD0201 cells cultured in BCELC differentiation medium without retinoic acid (RA) as supplement showed that the resulting monolayers fail to establish a proper barrier and that RepSox did not change this. Median with IQR. *n* = 6 individual filters. ns, not significant, determined by Mann-Whitney test. **(B)** Confocal overviews for Cldn6 immunostaining of BCELC monolayers treated with indicated conditions. Clear junctional signals were obtained for all conditions. **(C)** Cldn5, Cldn4, Cldn6 and ZO1 intensities (photon count) in STED images of junctional region. Cldn5 and Cldn6 intensities were reduced for 10 min and 60 min mannitol as well as for cCPE-SSS treatment compared to respective control. Cldn4 intensity was increased for 10 min mannitol. This may be related to a short-term compensatory liberation of Cldn4 from sequestered, non-junctional pools after hyperosmolaric stress [1]. Median with IQR. Cldn5 $n \geq 48$, Cldn4 $n \geq 18$, Cldn6 $n \geq 18$, ZO1 $n \geq 10$ with each *n* representing mean photon count of a single STED image in the junctional area. Adjusted P-values were determined with Kruskal-Wallis test followed by post-hoc Dunn's-Test with multiple comparison adjusted by using Holm-method. *p < 0.05, ***p < 0.0005, ****p < 0.0001.

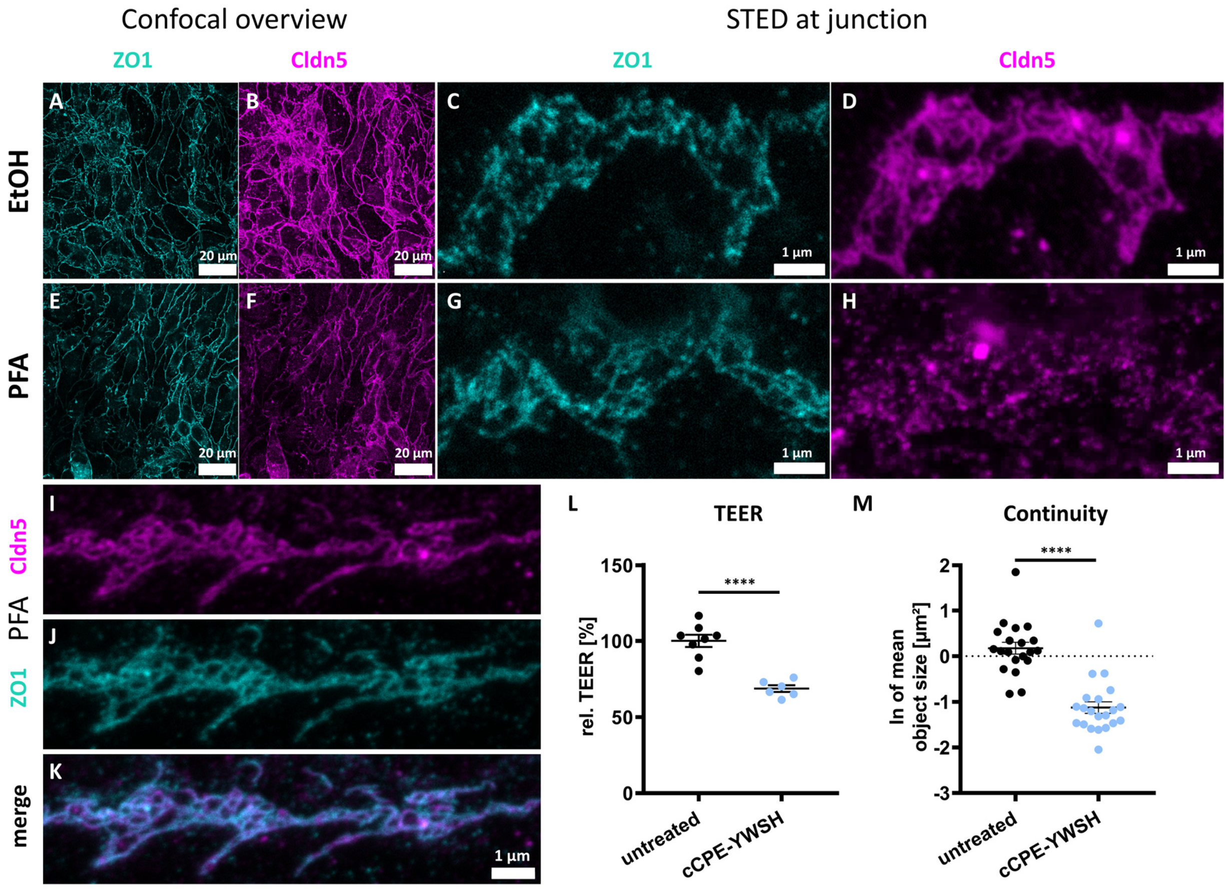

Figure S2 STED analysis was also performed with the BBB *in vitro* model cell line cerebEND [2, 3]. In these cells grown on transwell filters, ZO1 and Cldn5 were detected at cell-cell junctions after immunostaining (with mouse-anti-ZO1, rabbit-anti-Cldn5). (**A, B, E, F**) Confocal overviews for monolayers fixed with ethanol (EtOH) or 4% paraformaldehyde (PFA). (**C, D, G-K**) Using STED imaging at cell-junctional regions, meshworks of TJ strands containing Cldn5 and associated with ZO1 were resolved. However, EtOH- as well as PFA-fixed cells showed a strong heterogeneity with respect to cell morphology and the appearance of junctional structures. (**L**) TEER measurements of cerebEND monolayers. As reported previously [2, 3], cerebEND monolayers form a diffusion barrier that can be weakened by treatment with 10 µg/ml of the Cldn5 binder cCPE-Y306W/S313H (cCPE-YWSH). (**M**) Quantitative analysis of STED images showed a reduction of strand continuity (measured as decrease in strand object size) by the Cldn5 binder cCPE-YWSH. This disturbance of the meshwork integrity is suggested to contribute to the cCPE-mediated barrier disturbance.

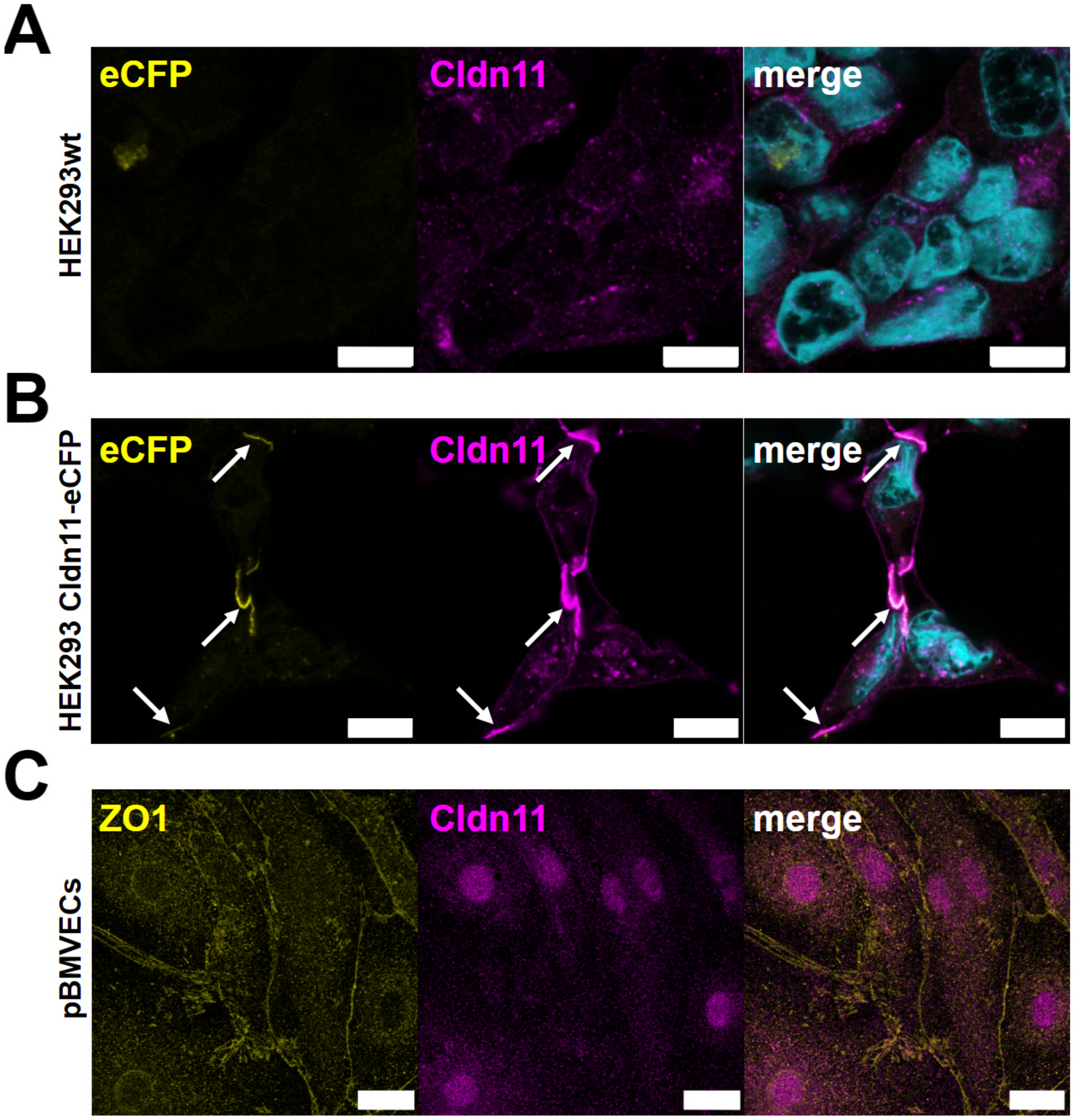

Figure S3. **Confocal imaging of Cldn11 in Hek293 cells and human primary brain microvascular endothelial cells (pBMVECs)** confirm the specificity and sensitivity of the anti-Cldn11 (#00211, BiCell) antibody, Hek293 cells without endogenous claudins were used. Non-transfected (wt) or Cldn11-transfected cells were cultured on glass coverslips and immunostained. **(A)** Hek293 wt cells showed some background signals but no enrichment of Cldn11 signal at cell-cell contacts. **(B)** Cldn11-eCFP-transfected Hek293 cells showed an enrichment of eCFP signal at cell-cell contacts. The anti-Cldn11 signal showed also a strong enrichment at cell-cell contacts coinciding with the eCFP signal (white arrows), showing that the antibody detects Cldn11. Merges include DAPI (cyan). **(C)** The tested anti-Cldn11 antibody was used to detect Cldn11 in pBMVECs cultured on Transwell inserts. No clear junctional Cldn11 signals but only presumably unspecific nuclear signals were obtained using the anti-Cldn11 antibody, similar to the unspecific intracellular signal observed in Hek293 wt cells shown in (A). As comparison, junctional signals were obtained for the ZO-1 counterstain (mouse anti-ZO1, #39-9100, Invitrogen Thermo Fisher Scientific Inc.). Scale bars, 10 µm.

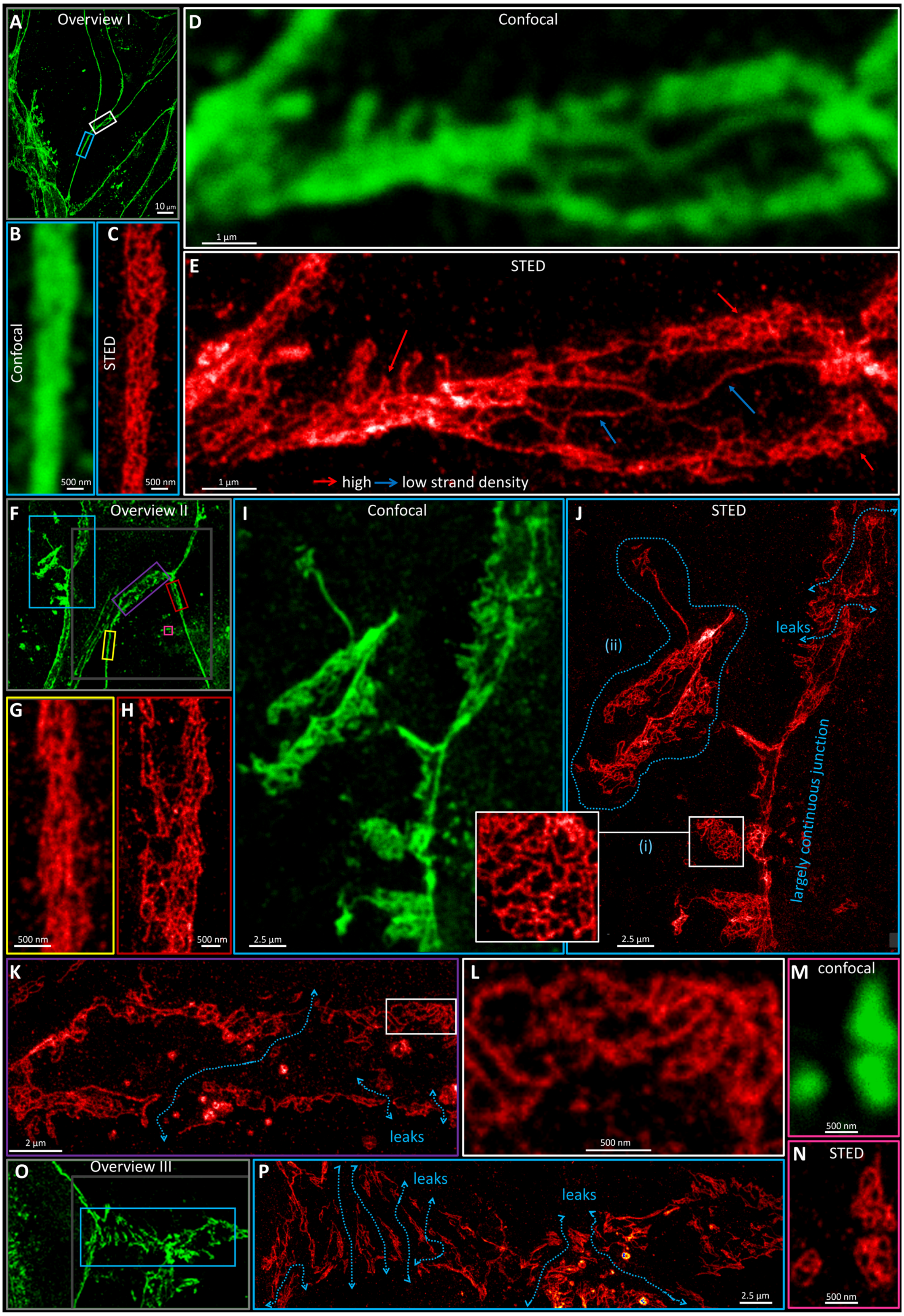
A
Overview I
10 µm
B
Confocal
500 nm
C
STED
500 nm
D
Confocal
1 µm
E
STED
1 µm
high
low strand density
F
Overview II
G
500 nm
H
500 nm
I
Confocal
2.5 µm
J
STED
(ii)
leaks
(i)
largely continuous junction
2.5 µm
K
leaks
2 µm
L
500 nm
M
confocal
500 nm
N
STED
500 nm
O
Overview III
P
leaks
leaks
2.5 µm

Figure S4. **Example confocal and STED images of pBMVECs cultured on Transwell inserts showing the spectrum of junctional Cldn5 structures that were found**. Cells were stained against Cldn5. Confocal images are shown in green and STED images in red. **(A)** Confocal overview I of monolayer showing continuous Cldn5 signals at most of the cell-cell contacts. **(B)** Confocal and **(C)** STED close-up of blue box in (A). Elongated junctional meshwork of continuous strands was resolved by STED but not by confocal imaging. **(D)** Confocal and **(E)** STED close-up of white box in (A). Junctional area with continuous meshwork of strands. Subareas with high and low strand densities could be distinguished. In both regions a continuous barrier was formed by multiple strands **(F)** Confocal Overview II. **(G, H)** STED close-ups of yellow and red boxes in (F) showing meshworks with slightly different width, strand density and continuity. **(I)** Confocal and **(J)** STED close-up of blue box in (F). Details were resolved by STED. Areas with breaks in the strand meshworks indicative for paracellular leaks (dashed double arrows) were found. In addition, meshworks without clear contribution to paracellular barrier were detected: (i) Pronounced meshwork extensions perpendicular to the junctional axis (with close-up in white box); (ii) big meshworks without connection to the main cell-cell contact (dashed encircled area). **(K)** STED close-up of purple box in (F). Junctional area with low strand density in center and leaks (dashed double arrows) due to discontinuities in the stand meshwork next to continuous parts of the meshwork (withe box, **(L)** close up). **(M)** Confocal and **(N)** STED close-ups of pink box in (F) showing structures that we speculated to be vesicles with internalized strands. **(O)** Confocal overview III. **(P)** STED close-up of blue box in (O) showing a junctional area with many breaks in the meshwork indicative for paracellular leaks (dashed double arrows).

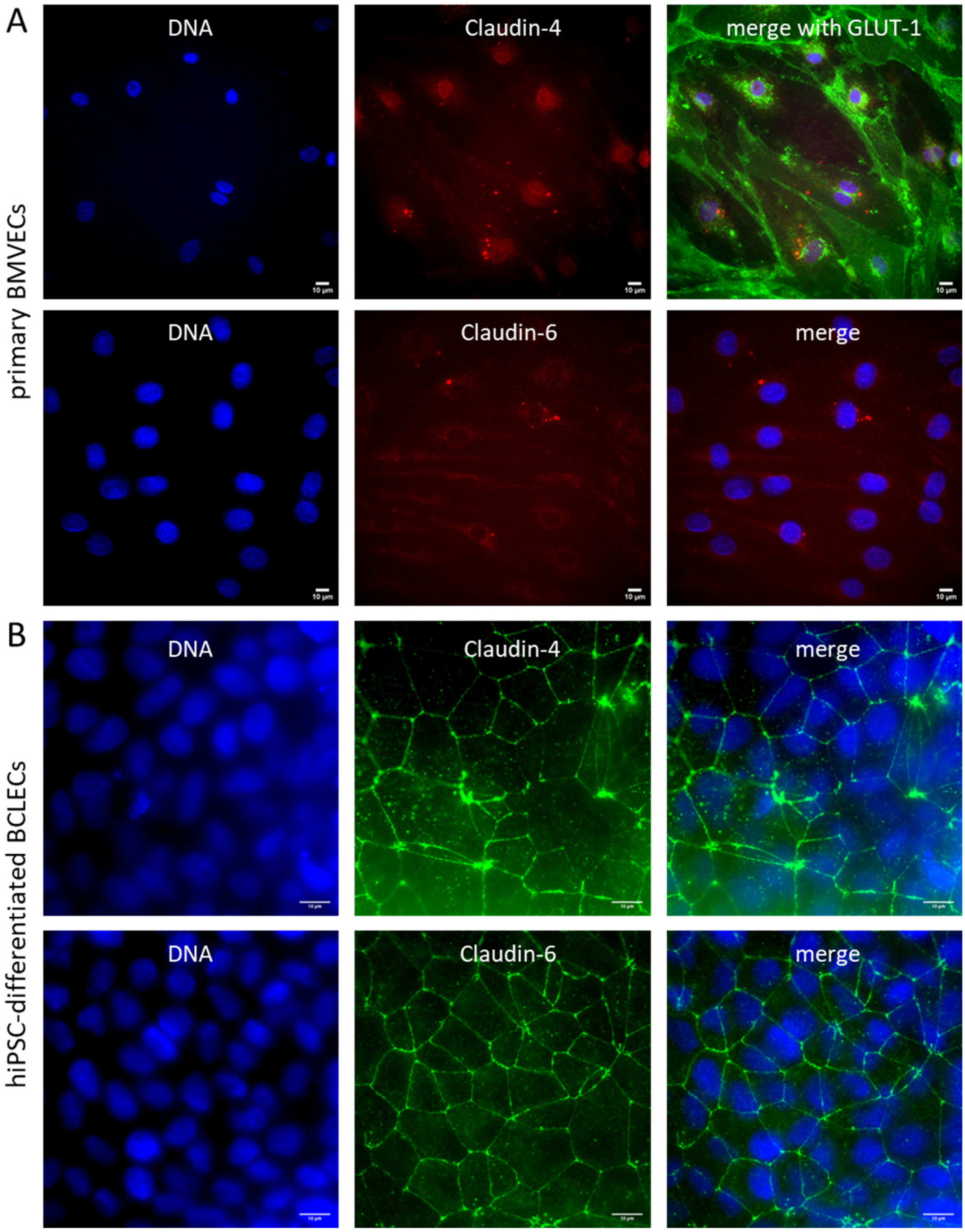

Figure S5. Immunostaining of Cldn4 and Cldn6 in pBMVECs **(A)** and hiPSC-differentiated BCLECs **(B)**. In contrast to SBAD0201-derived BCELCs, in pBMVEC only weak signals and no junctional localization was observed. Primary brain endothelial cells, fixed with EtOH, were co-stained with mouse anti-Claudin-4 (Invitrogen #32-9400) and rabbit anti-GLUT1 (Sigma-Aldrich #07-1401) or with rabbit anti-Claudin-6 (BiCell Scientific #00206). SBAD0201 hiPSC were differentiated to BCLECs, fixed with 4% PFA and permeabilized with 0.5% Triton-X100, and stained with rabbit anti-Claudin-4 (BiCell Scientific #00204) or rabbit anti-Claudin-6 (BiCell Scientific #00206). All samples were counterstained with DAPI and embedded in EverBrite™ Hardset Mounting Medium (Biotium) and imaged on an Olympus IX83 widefield epifluorescence microscope using a 60x objective with oil immersion.

Table S1 Median with IQR of BCELCs with standard DMSO and RepSox conditions (Fig. 2 F).

Condition	DMSO	RepSox
TEER [Ω·cm²]	6247.1<br>+168.0<br>- 33.6	3848.4<br>+517.1<br>-289.0

Table S2 Medians with IQR of raw TEER values from BCELCs treated with Mannitol (1.4 M) or cCPE-SSS (50 µg/ml) (Fig. 3 D & E). The high IQRs result from variations among the different BCELC batches used. This is one reason why we normalized the final TEER to the initial TEER before treatment in the analysis shown in Figures 3D and 3E.

Condition	untreated	10 min Mannitol	60 min Mannitol	Control 6 h	cCPE-SSS 6h	Control 24h	cCPE-SSS 24 h
initial TEER [Ω·cm²]	1768.9<br>+ 1953.9<br>- 406.2	1816.0<br>+ 2315.0<br>- 465.0	2172.1<br>+ 2341.9<br>- 747.3	1328.1<br>+ 2470.3<br>- 258.0	1412.1<br>+ 2104.9<br>- 197.7	4724.8<br>+ 251.2<br>- 275.5	5045.7<br>+ 98.3<br>- 143.6
final TEER [Ω·cm²]	2108.3<br>+ 1886.6<br>- 820.5	1481.0<br>+ 301.5<br>- 760.3	475.4<br>+ 98.3<br>- 160.4	2020.9<br>+ 2320.1<br>- 743.1	25.1<br>+ 4.2<br>- 9.6	2725.6<br>+ 693.8<br>- 58.8	18.6<br>+ 2.2<br>- 2.7

Table S3 Means with SEM of the raw TEER values from pBMVECs treated with different cCPE biologics (25 µg/ml) (Fig. 5 C).

Condition	control	cCPE-YL	cCPE-SSS	cCPE-YWSH
initial TEER [Ω·cm²]	466 +/- 59	488 +/- 47	503 +/- 43	506 +/- 53
final TEER [Ω·cm²]	429 +/- 40	421 +/- 47	223 +/- 23	271 +/- 25

Table S4 Means with SEM of the raw TEER values from cerebEND cells treated with 10 µg/ml cCPE-YWSH (Fig. S2 L).

Condition	control	cCPE-YWSH
TEER [Ω·cm²]	46.4 +/- 1.9	32.0 +/- 0.8

References

1. Cho Y, Taniguchi A, Kubo A, Ikenouchi J: Rho-ROCK liberates sequestered claudin for rapid de novo tight junction formation. *Elife* 2025, 13.
2. Neuhaus W, Piontek A, Protze J, Eichner M, Mahringer A, Subileau EA, Lee IM, Schulzke JD, Krause G, Piontek J: Reversible opening of the blood-brain barrier by claudin-5-binding variants of Clostridium perfringens enterotoxin's claudin-binding domain. *Biomaterials* 2018, 161:129-143.
3. Silwedel C, Forster C: Differential susceptibility of cerebral and cerebellar murine brain microvascular endothelial cells to loss of barrier properties in response to inflammatory stimuli. *J Neuroimmunol* 2006, 179:37-45.
